# Cross-platform transcriptomic profiling of the response to recombinant human erythropoietin

**DOI:** 10.1038/s41598-021-00608-9

**Published:** 2021-11-04

**Authors:** Guan Wang, Traci Kitaoka, Ali Crawford, Qian Mao, Andrew Hesketh, Fergus M. Guppy, Garrett I. Ash, Jason Liu, Mark B. Gerstein, Yannis P. Pitsiladis

**Affiliations:** 1 School of Sport and Health Sciences, University of Brighton, Brighton, UK; 2 Centre for Regenerative Medicine and Devices, University of Brighton, Brighton, UK; 3 Illumina, San Diego, CA USA; 4 BGI, Shenzhen, China; 5 School of Applied Sciences, University of Brighton, Brighton, UK; 6 Centre for Stress and Age-Related Disease, University of Brighton, Brighton, UK; 7 Veterans Affairs Connecticut Healthcare System, West Haven, CT USA; 8 Center for Medical Informatics, Yale University, New Haven, CT USA; 9 Program in Computational Biology and Bioinformatics, Yale University, New Haven, CT USA; 10 Department of Molecular Biophysics and Biochemistry, Yale University, New Haven, CT USA; 11 Department of Computer Science, Yale University, New Haven, CT USA; 12 Department of Statistics and Data Science, Yale University, New Haven, CT USA

**Keywords:** Computational biology and bioinformatics, Molecular biology, Systems biology

## Abstract

RNA-seq has matured and become an important tool for studying RNA biology. Here we compared two RNA-seq (MGI DNBSEQ and Illumina NextSeq 500) and two microarray platforms (GeneChip Human Transcriptome Array 2.0 and Illumina Expression BeadChip) in healthy individuals administered recombinant human erythropoietin for transcriptome-wide quantification of differential gene expression. The results show that total RNA DNB-seq generated a multitude of target genes compared to other platforms. Pathway enrichment analyses revealed genes correlate to not only erythropoiesis and oxygen transport but also a wide range of other functions, such as tissue protection and immune regulation. This study provides a knowledge base of genes relevant to EPO biology through cross-platform comparisons and validation.

## Introduction

High-throughput technologies in gene discovery, quantification and functional investigation have advanced our understanding of complex traits and facilitated disease diagnosis, prevention and treatment over the past decade^1,2^. Although the technologies continue to evolve for characterising genes and discerning gene-protein interactions both *ex-* and *in-vivo*, RNA-seq of bulk cells can directly capture global gene expression patterns underpinning important biological processes. Which tools to use will depend on the research question. Here, we performed gene expression profiling using two RNA-seq and two microarray platforms in healthy individuals administered recombinant human erythropoietin (rHuEPO). This study is the first of its kind investigating transcriptome-wide responses to rHuEPO in healthy individuals using RNA-seq. It differs from previous reports in (1) systematic comparisons of the MGI DNBSEQ-G400RS and Illumina NextSeq 500, (2) comparisons with the benchmarking GeneChip Human Transcriptome Array 2.0 and Illumina HumanHT-12 v4 Expression BeadChip, (3) emphasis on experimental and bioinformatics techniques to reducing technical variations and tackling artefacts, (4) use of a relatively large number of the same experimental samples across the platforms, and (5) adoption of a data triangulation approach to prioritising genes of biological relevance.

EPO is a glycoprotein hormone, mainly synthesised by the kidneys, but also by the liver. EPO secretion leads to red blood cell production (erythropoiesis). However, EPO also plays critical roles in tissue protection through direct effects on the immune cells, such as macrophages and lymphocytes, thus being a promising drug target for autoimmune diseases, allergies, reoxygenation injury and organ transplantation (reviewed in Ref^3^). The clinical usage of EPO in anticancer treatments is, however, controversial and should be applied with caution^3^. In addition, transcriptional regulation of the *EPO* gene is highly sensitive to activation of the hypoxia-inducible factor (HIF) pathway by the HIF prolyl hydroxylase inhibitors, which pose therapeutic potential for renal anaemia^4^. The erythropoiesis-stimulating property of EPO also means that it can be manipulated to gain advantage in sporting events, such as long distance running and rowing. For this reason, its use in sport has been banned since the early 1990s. Current direct and indirect detection methods focus on changes in the charge profiling of endogenously and exogenously produced EPO in urine^5^ and on abnormal variations in haematological parameters, for instance, the longitudinal follow-up of reticulocyte and haemoglobin level using the so-called Athlete Biological Passport^6^. Despite these achievements, refining and developing strategies with improved sensitivity to deter the misuse of EPO is an ongoing endeavour being facilitated by the World Anti-Doping Agency.

Specifically, we recruited 18 endurance-trained Caucasian males at sea level (Glasgow, Scotland; age: 26.0 ± 4.5 years, weight: 74.8 ± 7.9 kg, height: 179.8 ± 5.4 cm), who underwent 4 weeks of rHuEPO injections (50 IU/kg every 2 days)^7^. Whole blood samples were collected from the 18 subjects across the 8 time points: − 14- and − 1-day prior to the first injection (Base1 and Base2); 2-, 14- and 28-day following the first injection (EPO3, EPO4 and EPO5); and 7-, 14- and 28-day after the last injection (Post6, Post7 and Post8). Gene expression was measured by the Illumina HumanHT-12 v4 Expression BeadChip previously^8^ (Table 1). In the current study, 50 samples from 10 of the 18 subjects across Base1, Base2, EPO3, EPO4 and Post7 were randomly selected for gene expression profiling on the MGI and Illumina RNA-seq platforms as well as on the GeneChip (Table 1). This study aimed to determine the transcriptome-wide responses to rHuEPO in healthy individuals by cross-platform comparisons and create a knowledge base of genes implicated in EPO biology, thus informing potential therapeutic strategies for nephrology and immune diseases as well as facilitating the development of robust gene signatures for tackling blood doping in sport.

**Table 1:** Experimental time points, array and RNA-seq platforms and number of individuals analysed in the study.

Days	Time point	Arrays (N)	RNA-seq (N)
− 14	Base1	HT12v4 (18); HTA2.0 (10)	Illumina (10); MGI (10)
− 1	Base2	HT12v4 (18); HTA2.0 (10)	Illumina (10); MGI (10)
2	EPO3	HT12v4 (18); HTA2.0 (10)	Illumina (10); MGI (10)
14	EPO4	HT12v4 (18); HTA2.0 (10)	Illumina (10); MGI (10)
28	EPO5	HT12v4 (18); –	–
+ 7	Post6	HT12v4 (18); –	–
+ 14	Post7	HT12v4 (18); HTA2.0 (10)	Illumina (10); MGI (10)
+ 28	Post8	HT12v4 (18); –	–

Samples collected across all eight time points were previously subjected to analysis on the HT12v4, and the raw intensities were re-analysed in the current study. Base1 severed as the baseline for gene expression comparisons. N: the number of individuals. HT12v4: illumina HumanHT-12 v4 expression BeadChip; HTA2.0: GeneChip HTA2.0; Illumina: Illumina NextSeq 500; MGI: MGI DNBSEQ-G400RS. –: not applicable.

## Results

### Cross-platform differential gene expression analyses uncovered a treasure trove of genes significantly expressed following the rHuEPO administration

We identified 16,738 genes (MGI RNA-seq), 16,581 genes (Illumina RNA-seq), 29,517 transcript clusters (GeneChip), and 10,622 transcripts (BeadChip) for the differential gene expression (DGE) analyses (Table 2). The MGI and Illumina RNA-seq platforms produced good base call quality (quality score > 34, Supplementary Fig. S1). No sample contamination/swaps (Supplementary Fig. S2) and no other significant surrogate variables of batch effects were detected in these sequencing datasets. Genome mapping using HISAT2^9^ (against the reference genome assembly GRCh38.p12)^10^ showed the overall alignment rates of 94.2% (MGI; 197.9 M reads) and 95.0% (Illumina; 110.4 M reads) (Supplementary Table S1). RseQC^11^ revealed a large proportion of sequences aligned to introns in the MGI RNA-seq data (37.4%, versus 8.7% Illumina on average; Supplementary Fig. S3 and Supplementary Table S2), coinciding with the differences originating from sequencing library preparation (total RNA-seq with rRNA depletion and globin mRNA reduction, MGI versus mRNA enrichment, Illumina). RseQC also showed that 52.4% (MGI; ~ 97.8 M reads) and 74.8% (Illumina; ~ 78.2 M reads) were effectively mapped to the coding sequences (exons) (Supplementary Table S2 The average Salmon^12^ transcriptome mapping rates were 38.8% (MGI; 38.3 M reads) and 81.9% (Illumina; 45.1 M reads) (Supplementary Table S3). The discrepancies observed in reads and alignment rates across the software tools (HISAT2, RseQC and Salmon) were expected given their specific usage. Overall, the data suggest high quality sequences generated by both platforms. For the purposes of cross-platform comparison, the relative abundance estimates of transcripts following the Salmon mapping were summed to gene level, and genes were considered expressed when the gene-level abundance estimates were equal to or more than 5 in at least 4 samples. It led to the exclusion of 17,198 (MGI) and 18,347 (Illumina) genes (Table 2). Furthermore, 3852 (MGI) and 2860 (Illumina) un-defined gene mappings were removed following annotation (Table 2). Finally, 16,738 (MGI) and 16,581(Illumina) protein-coding genes were available for the downstream DGE analysis (Table 2).

**Table 2:** Transcript annotation and filtering of the RNA-seq and microarray data prior to the DGE analysis.

	MGI DNBSEQ-G400RS	Illumina NextSeq 500	GeneChip HTA2.0	Illumina HumanHT-12 v4 Expression BeadChip
Annotation database (N = the number of transcriptomic features)	Org.Hs.eg.db (N = 175,775 transcripts following Salmon transcription quantification, aggregated into 37,788 genes using Ensembl 94 annotation)	Org.Hs.eg.db (N = 175,775 transcripts following Salmon transcription quantification, aggregated into 37,788 genes using Ensembl 94 annotation)	hta20transcriptcluster.db (N = 285,543 transcripts, corresponding to 67,480 protein-coding and non-protein coding transcript clusters)	IlluminaHumanv4.db (N = 47,286 coding and non-coding transcripts)
Undetected probes	–	–	–	18,494
Low quality probes	–	–	–	6900
Low-expressed genes (RNA-seq)	17,198	18,347	–	–
“NA” mapping to stable gene symbols	3675	2668	36,709	2406
Mapping to multiple stable gene symbols	177	192	1254	698
Low-expressed probes (microarray)^a^	–	–	0	8166
Identified features available for DGE analysis	16,738^ g^	16,581^ g^	29,517^tc^	10,622^t^

NA: features with no gene symbols returned after annotation. DGE: differential gene expression. –: not applicable.

^a^Low expressed probes were further removed following assessing the average log expression and the mean–variance relationship after fitting the linear model in limma microarray analysis. g: protein-coding gene features. tc: protein-coding and non-protein coding transcript clusters, loosely corresponding to genes. t: coding and non-coding transcript features.

Initial quality control metrics revealed variability in eight out of the fifty GeneChip arrays (Supplementary Fig. S4). Two of the eight samples were then repeated for chip scanning, and the other six samples were repeated from the target preparation step (Supplementary Fig. S4). For consistency and completeness, we re-analysed the raw expression data of all samples available from the BeadChip arrays in the current study. The raw intensity values corresponded to 67,480 (GeneChip) and 47,286 (BeadChip) coding and non-coding transcriptomic features (Table 2). Subsequent data normalisation and filtering unveiled 29,517 transcript clusters (GeneChip) and 10,622 transcripts (BeadChip) for DGE analyses (Table 2). Briefly, 18,494 and 6900 probes were removed as undetected and low-quality probes, respectively, from the BeadChip (Table 2). While 8166 probes were removed due to low average expression (cutoff value: 5.1) in the BeadChip data, no probes of low expression were excluded from the GeneChip (Table 2 and Supplementary Fig. S5). No significant surrogate variables representing underlying biases, which may arise from library preparation and/or scanning, were observed in the two microarrays.

**Figure 1:**
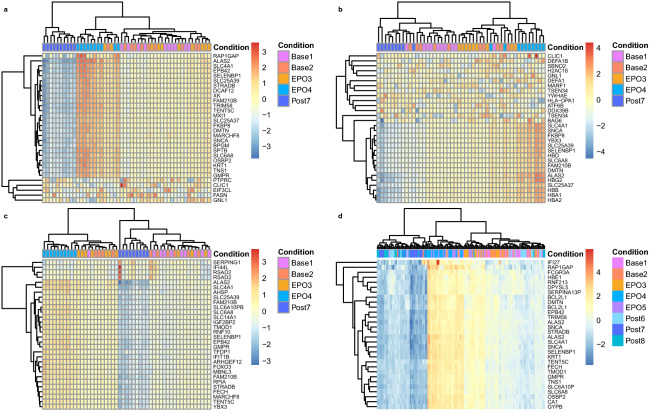
Gene clustering analysis of the top 30 genes of high variance across the RNA-seq and the microarray platforms. (**a**) MGI DNBSEQ-G400RS, (**b**) Illumina NextSeq 500; (**c**) GeneChip HTA2.0; (**d**) Illumina HumanHT-12 v4 Expression BeadChip.

**Figure 2:**
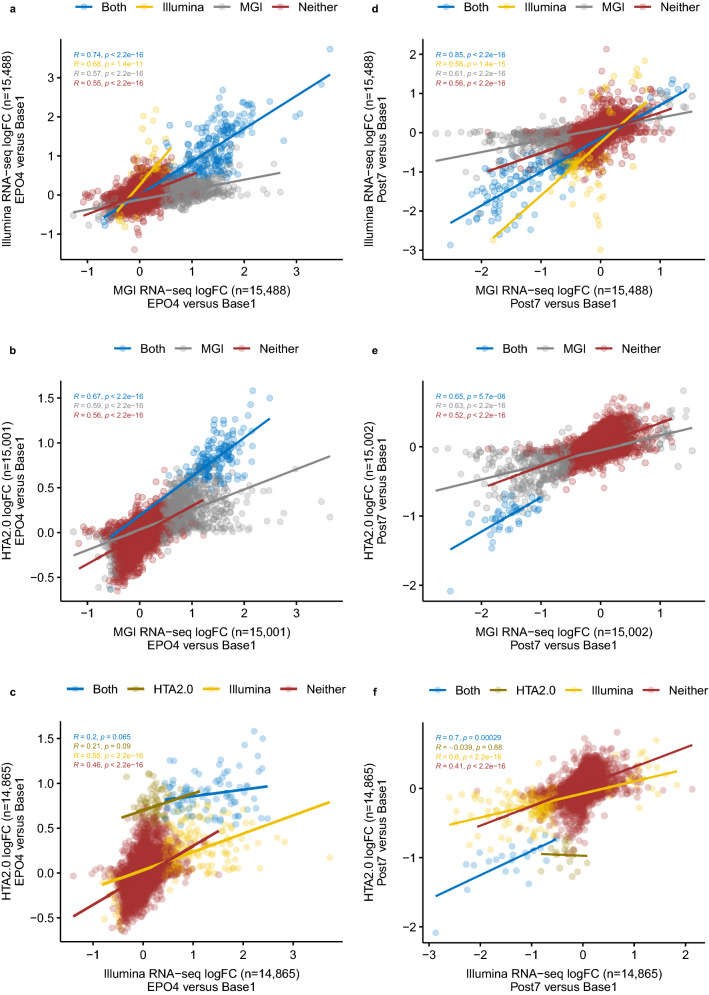
Cross-platform gene expression correlation analyses of log_2_-transformed fold changes of all identified gene features. (**a**–**c**) Genes identified when compared the level of expression between EPO4 and Base1 among the platform pairs of Illumina-MGI RNA-seq (**a**), GeneChip HTA2.0-MGI RNA-seq (**b**), GeneChip HTA2.0-Illlumina RNA-seq (**c**). **d-f** Genes identified when compared the level of expression between Post7 and Base1 among the platform pairs of Illumina-MGI RNA-seq (**d**), GeneChip HTA2.0-MGI RNA-seq (**e**), GeneChip HTA2.0-Illlumina RNA-seq (**f**). Only protein-coding genes are included here for cross-platform comparisons. Genes identified as differentially expressed by each pair are plotted in blue; genes that are only differentially expressed in Illumina RNA-seq, MGI RNA-seq or GeneChip HTA2.0 are plotted in yellow, grey and dijon, respectively; genes not identified as differentially expressed by a pair are plotted in red. For simplicity, the maximum expression value of a gene was used when multiple transcript cluster IDs or Ensembl gene IDs were mapped to the same gene symbol. *FOXO3B* is only differentially expressed in GeneChip HTA2.0 when compared to the MGI RNA-seq findings in (**b**), thus it has been removed from the correlation analysis. R: Pearson’s *r*. LogFC: log_2_-transformed fold change.

Unsupervised principal component analysis (PCA) revealed substantial variance, estimated using the top 500 genes ranked by expression variance across all samples. Variances explained by the principal component 1 (PC1) and principal component 2 (PC2) were: 69% versus 5% (MGI), 44% versus 9% (Illumina), 58% versus 14% (GeneChip), and 78% versus 7% (Beadchip) (Supplementary Fig. S6). Gene clustering of the top 30 genes of high variance showed a good distinction across the time points in all datasets (Fig. 1). Nevertheless, a more distinctive pattern was observed following MGI RNA-seq compared to Illumina RNA-seq and GeneChip (Fig. 1a vs b,c). Despite more samples were analysed using the BeadChip in 18 subjects, higher variances of the top 30 genes were found following MGI RNA-seq (Fig. 1a vs d). The DESeq2^13^ and limma^14^ DGE analyses yielded 1552 genes (MGI), 582 genes (Illumina), 252 transcript clusters (GeneChip) and 2372 transcripts (BeadChip), exceeding the pre-defined thresholds (RNA-seq: fold change of 1.2 and *s *value of 0.005; microarray: fold change of 1.2 and BH adjusted *p *value of 0.05; note that the probability thresholds bound to the fold change of 1.2) (Table 3). Among the coding genes characterised by the MGI and Illumina RNA-seq and GeneChip, a significant proportion was unique to MGI RNA-seq (66.8% at EPO4 and 54.5% at Post7) (Supplementary Table S4). Substantial proportions of these MGI genes exceeded an absolute fold change of 2 (12.4% at EPO4 and 18.0% at Post7) as well as captured moderate changes between 1.2 and 2 (54.4% at EPO4 and 36.5% at Post7), when compared to the coding genes determined by Illumina RNA-seq and GeneChip (ranging from 0 to 19.4%) (Supplementary Fig. S7 and Supplementary Table S4). Furthermore, strong correlations were observed between the MGI and Illumina RNA-seq platforms (*r* = 0.74 at EPO4 and *r* = 0.85 at Post7, *P* < 2E−16; Fig. 2a,d), whereas the correlations ranged from very weak (*r* = 0.2) to moderate (*r* = 0.7) when compared RNA-seq to GeneChip (*P* < 0.0003; Fig. 2b,c,e,f). Overall, the MGI total RNA DNB-seq resulted in an increased sensitivity in detecting differential gene expression in healthy individuals following the rHuEPO administration compared to the Illumina mRNA-seq and GeneChip (Fig. 2, Supplementary Fig. S7 and Supplementary Data 1).

**Table 3:** Summary of the number of significantly expressed transcriptomic features across the platforms.

	DGE thresholds	Base2 versus base1	EPO3 versus base1	EPO4 versus base1	Post7 versus base1	Up/down regulation
MGI DNBSEQ-G400RS (N = 50)	abs FC > 1.2 and *s *value < 0.005	0	1	959	60	Up
		0	0	81	451	Down
Illumina NextSeq 500 (N = 48)	abs FC > 1.2 & *s *value < 0.005	0	0	277	27	Up
		0	0	20	258	Down
GeneChip HTA2.0 (N = 49)	abs FC > 1.2 and FDR < 0.05	0	0	200	0	Up
		0	0	1	51	Down
Illumina HumanHT-12v4.0 Expression Beadchip (N = 143)	abs FC > 1.2 and FDR < 0.05	0	13	796	7	Up
		0	0	1315	254	Down

N: the number of samples. DGE: differential gene expression. abs FC: absolute fold change. FDR: false discovery rate. Two, one and one samples were removed from the DGE analyses due to human processing errors, sample quality and sampling issue in the Illumina RNA-seq, GeneChip and BeadChip datasets, respectively. The number of protein-coding gene features, and coding and non-coding transcript clusters and transcripts are reported following the RNA-seq, GeneChip and Beadchip DGE analyses, respectively. Note, for the Illumina BeadChip, only DGE results corresponding to the same time points as the other three platforms are provided in the table above.

### Functional enrichment analyses unveiled genes linked to erythropoiesis as well as non-erythropoietic functions of EPO

To explore the biological functions of the transcriptomic features identified from sequencing and microarray, we performed a standard GSEA run (v4.0.3) subjected to 1000 phenotype permutations^15,16^ for all datasets, using the MSigDB (v7.2)^15,17^ hallmark (H)^18^ and Gene Ontology (C5; BP: GO biological process)^19,20^ collections of functional gene sets. As expected, heme metabolism emerged as the most significantly enriched gene set following the analysis of 50 hallmark gene sets (FDR: MGI = 0.011 EPO4, Illumina = 0.033 EPO4, GeneChip ≤ 0.017 EPO4/Post7 and BeadChip ≤ 0.004 EPO3/4/5/Post7/8; Supplementary Table S5).This observation is in line with the changes in reticulocyte and haematocrit during and post rHuEPO administration in the same cohort of samples, previously reported^7,8^. Leading edge genes included 144 (MGI; EPO4), 105 (Illumina; EPO4), 125/96 (GeneChip; EPO4/Post7) and 101/103/103/97/84 genes (BeadChip; EPO3/4/5/Post7/8), contributing to the heme metabolism enrichment scores (Supplementary Data 2). Fifty-six leading edge genes overlapped across all platforms and across the time points (Supplementary Data 2). Of the 56 genes, 51 and 34 genes were differentially expressed at EPO4 and Post7, respectively, across the RNA-seq and GeneChip platforms (Supplementary Data 3). GSEA was able to detect genes that have fallen off the DGE detection thresholds in the Illumina RNA-seq and GeneChip datasets (Supplementary Data 3). In addition, 10, 51, 51, 36, and 19 of the 56 leading edge genes overlapped with the BeadChip DGE genes at EPO3, EPO4, EPO5, Post7 and Post8, respectively (Supplementary Data 4). The data suggest the effectiveness of all four platforms and the effectiveness of GSEA in capturing the most context-relevant biological pathway relevant to EPO biology.

**Figure 3:**
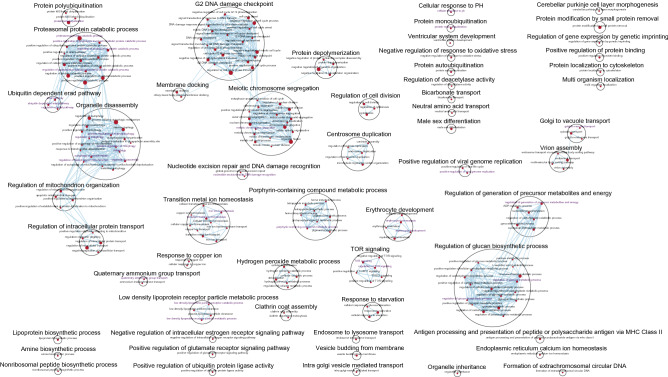
Biological network of the MGI RNA-seq dataset following Gene Ontology (biological process) gene set enrichment analysis in GSEA (v4.0.3) and visualisation in Cytoscape (3.8.0). Each circle (node) represents a gene set and two nodes are connected by lines (edges) indicating shared genes. The size of a node and width of an edge are proportional to the number of genes enriched in a gene set and the number of genes shared between gene sets, respectively. Gene sets that are similar were annotated and clustered to form a biological theme using the AutoAnnotate App in Cytoscape. The most significantly enriched gene set is used to label a gene set cluster, defined by NES. Red node: gene set enriched in EPO4. Purple node label: top gene sets with NES > 1.90. The enrichment map was created with pathway FDR < 0.1, nominal *P* < 0.05 and Jaccard Overlap coefficient > 0.375 with combined constant k = 0.5.

Next, GSEA was conducted using 7530 GO biological processes in the MSigDB C5 collection, and identified a total of 212, 134, and 33 biological pathways enriched in the MGI RNA-seq (EPO4), GeneChip (EPO4) and BeadChip (EPO4, EPO5, Post7 and Post8) datasets, respectively (pathway FDR < 0.1 and nominal *P* < 0.05). No significantly enriched GO biological processes were identified in the Illumina RNA-seq datasets. For the MGI RNA-seq EPO4 dataset, these included biological processes resembling EPO cytoprotective functions and the downstream signal transduction pathways^21–23^; typically involved in response to oxidative stress (e.g. positive regulation of mitophagy, hydrogen peroxide metabolic process, and nucleotide-excision repair, DNA damage recognition), heme formation, erythrocyte development, mechanistic target of rapamycin (mTOR) signalling, regulation of energy metabolism, low density lipoprotein clearance, and nervous system development (Fig. 3). Pathways consisting of autophagy of mitochondrion, positive regulation of cell cycle arrest, iron ion homeostasis, tetrapyrrole metabolic process, erythrocyte development, and ventricular system development were found in the GeneChip EPO4 dataset (Supplementary Fig. S8). In addition, other biological processes, such as cyclic GMP mediated signalling, positive regulation of cardiac muscle cell proliferation, and gamma-aminobutyric acid transport, were also observed in this dataset (Supplementary Fig. S8). In the BeadChip datasets, the pathways common to those captured by the other platforms comprised hemoglobin metabolic process, erythrocyte development, and hydrogen peroxide metabolic process (Supplementary Fig. S9). Other pathways enriched in the BeadChip datasets included the negative regulation of necrotic cell death, negative regulation of TORC1 signalling, cellular response to monoamine stimulus, monoamine transport, gas transport, lipid transport, drug transmembrane transport, synaptic signalling, synapse organisation, and multicellular organism development (Supplementary Fig. S9).

Top 34, 14, and 16 pathways prioritised by the normalised enrichment score (NES) > 1.90 as well as 38, 66, and 12 pathways each representing a biological theme where NES < 1.90 were further examined in the MGI RNA-seq, GeneChip and BeadChip datasets, respectively. The leading edge genes were extracted from these pathways. In the MGI RNA-seq EPO4 dataset, 308 leading edge genes overlapped with the EPO4 DGE genes. Furthermore, 135 of the 308 genes were found to be significantly expressed at Post7 (Supplementary Data 5). Of the 135 genes, the top 10 genes according to GSEA ranks and pathway NESs (*BPGM*, *ALAS2*, *PKD1L3*, *SLC4A1*, *AP2A1*, *IGF2*, *FAM210B*, *DYRK3*, *FECH* and *SLC25A37*) are relevant to erythrocyte development, heme formation, metal ion homeostasis, cellular response to PH, LDL particle clearance, glucose and energy metabolism, and mTOR signalling (Supplementary Data 5). In the the GeneChip EPO4 dataset, 57 leading edge genes were common to the EPO4 DGE genes, while 15 (of the 57) were also present in the Post7 DGE results (Supplementary Data 6). The 15 genes, i.e. *ALAS2*, *SLC4A1*, *FOXO3*, *TMOD1*, *FECH*, *SLC6A8*, *SLC25A39*, *SNCA*, *FAM210B*, *EPB42*, *SLC25A37*, *YBX3*, *BPGM*, *STRADB*, and *BCL2L1*, are correlated with heme formation, bicarbonate transport, muscle atrophy, lens fiber cell development, gamma-aminobutyric acid transport, erythrocyte development, cellular hyperosmotic response, negative regulation of signal transduction in the absence of ligand and cellular response to amino acid stimulus (Supplementary Data 6). Among 376 leading edge genes identified from the BeadChip datasets, 76 were observed following the DGE analyses across EPO4, EPO5, Post7 and Post8. The top 10 genes (*KCNJ10*, *YBX3*, *SNCA*, *OR2W3*, *IRX1*, *OR2W5*, *CAMK2A*, *ACP4*, *NCDN* and *HOXC10*) are involved in regulation of neuronal synaptic plasticity and necrotic cell death, sensory perception of smell, proximal/distant pattern formation, and cell fate specification (Supplementary Data 7). Among the above 135, 15 and 76 genes, *BPGM*, *ALAS2*, *SLC4A1*, *FAM210B*, *EPB42*, *SNCA*, *YBX3* and *TMOD1* were detected by all three platforms (Supplementary Fig. S10 and Supplementary Data 8). *FECH*, *SLC25A37*, *FOXO3*, *BCL2L1*, and *SLC25A39* were common between MGI RNA-seq and GeneChip, *SLC6A8* between GeneChip and BeadChip, and *SLC7A5*, *PINK1*, *DMTN*, *TRIM58*, *SESN3*, *GATA1*, *FURIN*, *HBQ1*, *EIF2AK1*, and *HBM* between MGI RNA-seq and BeadChip (Supplementary Fig. S10 and Supplementary Data 8). One hundred and twelve leading edge genes (top 5: *PKD1L3*, *AP2A1*, *DYRK3*, *IGF2* and *TAL1*) were uniquely identified by MGI RNA-seq, 1 (*STRADB*) by GeneChip and 57 by BeadChip (top 5: *KCNJ10*, *OR2W3*, *IRX1*, *OR2W5* and *CAMK2A*) (Supplementary Fig. S10 and Supplementary Data 8). Furthermore, 43 genes identified from one or more of the three platforms were confirmed in the Illumina RNA-seq DGE analyses across EPO4 and Post7 (Supplementary Fig. S10 and Supplementary Data 9).

### Validated genes have the potential for targeted therapeutic interventions for blood and immune diseases and for combating blood doping in sport

**Figure 4:**
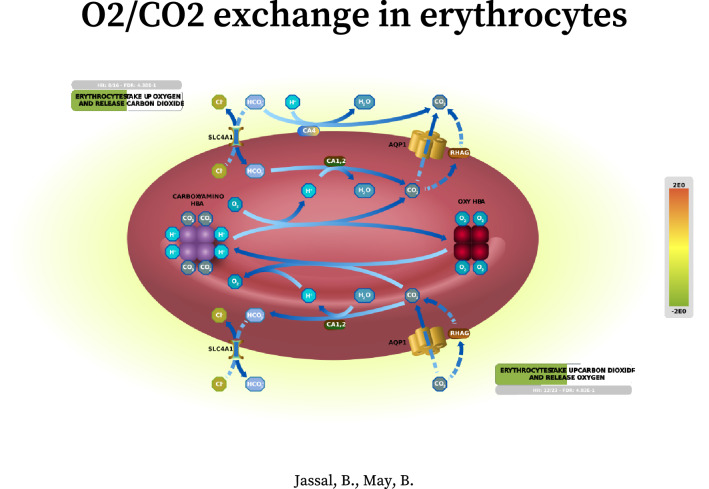
Enhanced high-level Reactome pathway diagram for O_2_/CO_2_ exchange in erythrocytes^59^ by expression overlay with the MGI RNA-seq Post7 dataset (credit: Jassal B and May B at European Bioinformatics Institute; New York University Langone Medical Center; Ontario Institute for Cancer Research; Oregon Health and Science University). This high-level diagram represents two subpathways, namely erythrocyte take up oxygen and release carbon dioxide and erythrocyte take up carbon dioxide and release oxygen. The green band indicates the proportion of the pathway that is represented in the MGI RNA-seq Post7 dataset, and the colour (green) represents the down-regulation of the pathway genes. The grey bar contains the information on the number of pathway entities in the query dataset, the total number of the pathway entities, and the FDR corrected over-representation probability.

**Figure 5:**
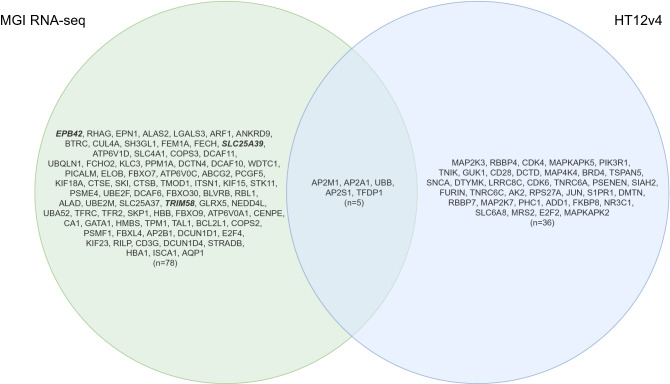
One hundred and nineteen genes confirmed following DGE, GSEA and Reactome analyses across the MGI RNA-seq and HT12v4 BeadChip platforms. *EPB42*, *SLC25A39* and *TRIM58* currently with no entries found in Reactome are highlighted in italics/bold. The diagram was drawn using the online open source tool, draw.io (v15.0.4; https://github.com/jgraph/drawio).

To follow up on these results, we performed additional analysis using the Reactome database to examine the pathway components inferred from the 43 genes in pathway diagrams and to establish a knowledge base of genes that are the most relevant to EPO biology. By overlaying gene expression values on Reactome pathway diagrams (release 73)^24^, 13 and 8 significantly expressed interaction networks represented by 29 and 13 genes (of the 43), or their interactors (IntAct score ≥ 0.556), were identified in the MGI RNA-seq and BeadChip datasets, respectively (pathway FDR < 0.05; see Supplementary Data 10 for pathway entities and statistics and Supplementary Data 11 for pathway overviews). Notably, pathway components in the entire cascade of O_2_/CO_2_ exchange in erythrocytes were the most significantly altered in the MGI RNA-seq datasets (subpathway FDR ranging from 0.00001 to 0.003, Supplementary Data 12), contrasting with the findings from Illumina RNA-seq, GeneChip and BeadChip datasets (subpathway FDR ranging from 0.00001 to 0.077; Supplementary Data 13–15). These included the pathway genes, *SLC4A1*, *HBB*, *CA1*, *AQP1*, *RHAG*, *HBA1* and *CYBSR1*, up-regulated at EPO4 and down-regulated at Post7 (see Fig. 4 for the enhanced high-level pathway diagram overlaid with the MGI Post7 gene expression values). Finally, by overlapping 172 (MGI) and 91 (BeadChip) significantly expressed Reactome pathway genes and their interactors of high confidence (IntAct score > 0.9, Supplementary Data 10) originated from the 13 and 8 networks with the DGE genes, 80 and 41 genes attributable to rHuEPO were further validated (Supplementary Data 16). In addition, we found no entries in Reactome for *EPB42*, *SLC25A39* and *TRIM58*, however, these genes are also considered validated for completeness. Together, a total of 119 genes represent the candidate genes that warrant further investigation for targeted therapeutics for blood diseases and immune regulatory dysfunction and for potential gene signatures of blood doping (Fig. 5). The subsets of the top 10 genes (sorted by the DGE *s *value < 0.005 or FDR < 0.05) along with the corresponding GSEA and Reactome pathways are presented in Supplementary Table S6 and S7. These comprise the top biological pathways enriched in mitochondrial iron-sulfur cluster biogenesis, metabolism of porphyrins, O_2_/CO_2_ exchange in erythrocytes, transcriptional activity of SMAD2/SMAD3:SMAD4 heterotrimer, MHC class II antigen presentation, Rhesus glycoproteins mediate ammonium transport, neddylation, creatine metabolism, potential therapeutics for SARS, oxidative stress induced cellular senescence, and amyloid fibre formation.

## Discussion

Taken together, we conducted direct cross-platform comparisons in the 10 subjects following differential gene expression analyses of rHuEPO across the MGI DNBSEQ-G400RS, Illumina NextSeq 500 and GeneChip HTA2.0 platforms. Given the large number of raw reads initially generated on the RNA-seq platforms (110.4 M Illumina mRNA-seq; 197.9 M MGI total RNA-seq; Supplementary Table S1), and despite no globin mRNA reduction carried out in the Illumina mRNA-seq, the alignment and quantification algorithms output a comparable number of exon sequences of transcripts from both RNA-seq platforms that were readily available for downstream DGE analysis (45.1 M Illumina; 38.3 M MGI; Supplementary Table S3). In addition, the large proportion of coding genes overlapped (n = 15,488; between 16,738 genes, MGI and 16,581 genes, Illumina) and strong correlations (e.g. *r* = 0.74 at EPO4 and *r* = 0.85 at Post7 for significantly expressed genes) between the RNA-seq platforms as well as the generally moderate-to-high correlations between the GeneChip and the RNA-seq platforms (Fig. 2) further support the validity of these results across the platforms. The data suggest no substantial biases in quantifying protein-coding genes, despite the differences in sample preparation/RNA-seq approaches. There was a 2.28-fold increase in the number of genes significantly expressed following MGI RNA-seq, compared to the combined number of genes revealed by Illumina RNA-seq and GeneChip platforms (Supplementary Fig. S7 and Supplementary Table S4). Specifically, among 1126 genes differentially expressed at EPO4, 25.5% of the genes overlapped between MGI RNA-seq and the other two platforms, and 66.8% of the genes were unique to MGI RNA-seq (Supplementary Table S4). Among 674 genes differentially expressed at Post7, the corresponding figures were 21.5% and 54.5%, respectively (Supplementary Table S4). Among genes with an absolute fold change less than 2, Illumina RNA-seq captured a much higher proportion of genes compared to GeneChip (10.3% vs 1.7% EPO4; 26.4% vs 0.6% Post7; Supplementary Table S4). The effect of EPO was largely captured by MGI RNA-seq (PC1: 69% vs PC2: 5%), followed by GeneChip (PC1: 58% vs PC2: 14%) and Illumina RNA-seq (PC1: 44% vs PC2: 9%) (Supplementary Fig. S6). These observations support the supreme performance of the MGI total RNA DNB-seq, followed by Illumina mRNA-seq and GeneChip HTA2.0, focusing on the protein-coding genes in this study. Nevertheless, genes characterised by the Illumina HumanHT-12 v4 Expression BeadChip in the 18 subjects represented a total of 85% of variance captured by PC1 (78%) and PC2 (7%) (Supplementary Fig. S6), reflecting the increased power due to the larger sample size.

Quantitative pathway analysis by GSEA unveiled that the MSigDB Hallmark gene set of heme metabolism was enriched across all four platforms (Supplementary Data 2). In addition, 212 (MGI), 134 (GeneChip), and 33 (BeadChip) enriched biological processes were identified. These results underpinned EPO biology, particularly with a wealth of functional information derived from the MGI RNA-seq (Fig. 3). Three hundred and eight, 57 and 376 leading edge genes were identified from these pathways prioritised by the NESs, subsequently leading to 135, 15 and 76 genes also confirmed by the DGE analyses (Supplementary Data 5–7). Among them, 43 were further validated in the Illumina RNA-seq DGE results (Supplementary Data 9). Despite strong positive correlations between the MGI and Illumina RNA-seq, the lack of significantly expressed pathways in the latter is in line with the weaker expression signals detected (Fig. 2).

To better understand the interacting networks and signalling cascades represented by the 43 genes, we explored the Reactome database and generated a total of 21 pathway overviews detailing the pathway entities, gene expression levels, and interactors (Supplementary Data 11). Notably, all four platforms detected significant changes in the signalling cascade of O_2_/CO_2_ exchange in erythrocytes, with the most significant changes uncovered by the MGI RNA-seq (Fig. 4); reiterating a strong effect of rHuEPO on oxygen uptake and delivery. Finally, by extracting the significantly altered genes in the Reactome networks and by matching them to the DGE genes, we concluded with 119 genes relevant to EPO biology originated from the MGI RNA-seq and BeadChip datasets (Fig. 5). These genes are found extensively in biological processes including heme biosynthesis and catabolism (*ALAS2, FECH, BLVRB, ALAD, HMBS, ABCG2,* and *ABCC2*) and in the aforementioned O_2_/CO_2_ exchange in erythrocytes (*SLC4A1*, *HBB*, *CA1*, *AQP1*, *RHAG*, *HBA1* and *CYBSR1*), which are central to erythropoiesis and oxygen transport. Other genes are involved in stimulating adaptive immune responses by MHC class II antigen presentation to CD4 + T helper cells (*RILP, KLC3, CTSE, ARF1, AP2A1, AP2M1, DCTN4, CENPE, DCTN4, KIF11, KIF15, KIF18A, KIF23, KIF3A, CTSB, AP2B1,* and *AP2S1*) and in Nef mediated downregulation of CD28 expressed on the surface of T cells (*AP2M1*), suggesting the direct effect of rHuEPO on the CD4 + cells in line with the other recent reports (reviewed in Ref^3^). *PSMF1, GATA1, TAL1, PSME4,* and *PCGF5* are implicated in transcriptional regulation by RUNX1, which is a master regulator of haematopoiesis^25^ and is often translocated in acute myeloid leukemia^26,27^. The RUNX1:CBFB heterodimer targets genes regulating self-renewal of haematopoietic stem cells and differentiation of haematopoietic progenitors, including myeloid, megakaryocytes, T cells and B cells^28^. *UBA52, UBB, PPM1A, NEDD4L, E2F4, TFDP1, RBL1, SKI, STRADB,* and *PPM1A* contribute to the transcriptional activity of the SMAD2/SMAD3:SMAD4 complex, a transcriptional regulator that controls the TGF beta signalling^29^. *STK11, STRADB,* and *PPM1A* modulate the formation of the LKB1:STRAD:MO25 complex and activation of AMPK by phosphorylation, which subsequently trigger the phosphorylation of TSC2 and inhibition of mTOR signalling^30^. However, this process can only be maintained when the cellular AMP:ATP ratio is high. In addition, the genes being prioritised in the current study are also involved in mitochondrial iron-sulfur cluster biogenesis (*SLC25A37, GLRX5,* and *ISCA1*), striated muscle contraction (*TPM1* and *TMOD1*), creatine metabolism (*SLC6A8*), interconversion of nucleotide di- and triphosphates (*GUK1, DTYMK, DCTD,* and *AK2*), oxidative stress induced senescence (*UBB, TNRC6C, MAP4K4, MAP2K3, E2F2, CDK4, TNIK, JUN, MAPKAPK2, PHC1, TFDP1, CDK6, TNRC6A, MAP2K7, MAPKAPK5, RPS27A,* and *RBBP7*), and tissue damage caused by amyloid deposition (*SIAH2, SNCA, TSPAN5, PSENEN, FURIN, UBB,* and *RPS27A*). These findings hold great promise for the development of effective therapeutic interventions targeting EPO and its signalling pathways. They also reflect the fact that the combination of the four gene expression profiling approaches with the use of the GSEA and MSigDB and Reactome databases to querying the data independently generated corroborating evidence across the platforms, supporting the validity and completeness of the results; thus, leading to a better understanding of EPO biology.

In conclusion, RNA-seq and microarrays applied here generated a robust set of genes relevant to the biology of EPO, particularly by the MGI total RNA DNB-seq. The adoption of a data triangulation approach reinforced the biological findings, which can be used to facilitate potential therapeutic interventions targeting EPO and its signalling pathways for treating blood and immune disorders as well as to establish additional gene signatures for tackling blood doping in sport.

## Methods

### Subjects

In a previously funded research project by the World Anti-Doping Agency (Grant No.: 08C19YP), we collected whole blood samples from 18 endurance-trained Caucasian males at sea level from Glasgow, Scotland (26.0 ± 4.5 years, 74.8 ± 7.9 kg, 179.8 ± 5.4 cm). They underwent 4-week 50 IU/kg body mass of rHuEPO injections every 2 days^7^. Daily oral iron supplementation (100 mg of elemental iron, ferrous sulphate tablets, Almus, Barnstable, UK) was given during the 4 weeks of rHuEPO administration^7^. Whole blood samples were collected at baseline (2 weeks and 1 day before rHuEPO; denoted by Base1 and Base2), during the rHuEPO administration (2 days, 2 and 4 weeks following the 1st injection; denoted by EPO3, EPO4 and EPO5) and for 4 weeks after the rHuEPO administration (1, 2 and 4 weeks after the last injection; denoted by Post6, Post7 and Post8) for gene expression profiling using the HumanHT-12 v4.0 Expression BeadChip (Illumina, San Diego, CA, USA)^8^. In the current study (grant no.: ISF15E10YP), samples from 10 out of the 18 subjects collected at Base1, Base2, EPO3, EPO4 and Post7 were analysed on a new microarray platform (the GeneChip Human Transcriptome Array 2.0 or the HTA2.0, Thermo Fisher Scientific, Waltham, MA, USA) and on two RNA-seq platforms (the NextSeq 500, Illumina, San Diego, CA, USA, and the DNBSEQ-G400RS, MGI Tech, Shenzhen, China) for cross-platform gene expression profiling in response to rHuEPO. The studies were approved by the University of Glasgow Ethics Committee (Scotland, UK) and the University of Brighton Ethics Committee (England, UK) and were performed in accordance with the “Declaration of Helsinki”. Written informed consent was obtained from all subjects.

### RNA collection and preparation

Three milliliters of whole blood was collected from an antecubital vein using the Tempus Blood RNA tubes (Thermo Fisher Scientific, Waltham, MA, USA). Each Tempus tube contains 6 mL of RNA stabilising reagent and was vigorously mixed immediately after collection for 10 s. The blood samples were incubated at room temperature for approximately 3 h (note that the gene expression profile of target genes is stable for up to 5 days at room temperature and for at least 1 week at 4 °C) and then stored at − 20 °C or − 80 °C before subsequent analysis or transportation to the analytical lab. Total RNA was isolated from the whole blood according to the manufacturer’s instructions (Tempus Spin RNA Isolation Kit, Thermo Fisher Scientific, Waltham, MA, USA). The purified total RNA was eluted in 90 μL elution buffer and stored in three aliquots at − 80 °C until further analysis. Initial RNA quantity and purity was assessed by the Nanodrop ND-2000 Spectrophotometer (Thermo Fisher Scientific, Waltham, MA, US). RNA integrity was assessed using the Agilent 2100 Bioanalyser (Agilent Technologies, Santa Clara, CA, USA) prior to the RNA-seq and GeneChip analyses.

### Microarray analysis with the HumanHT-12 v4.0 expression BeadChip

Detailed sample preparation for the Illumina microarray experiment is available elsewhere^8^. Briefly, 500 ng of total RNA was used for complimentary RNA (cRNA) synthesis using the Illumina TotalPrep RNA Amplification Kit (Thermo Fisher Scientific, Waltham, MA, USA). Seven hundred and fifty nanograms of the purified labelled cRNA samples were hybridised to the HumanHT-12 v4.0 Expression BeadChip arrays containing > 47,000 probes, following the manufacturer’s recommended procedures (Illumina, San Diego, CA, USA). The Bead arrays were scanned on the Illumina BeadArray Reader. In the current study, the raw intensity values were exported using the Illumina GenomeStudio software (v2.0; Gene Expression Module) and were re-analysed in order to standardise and optimise data processing for comparisons with the other platforms. Specifically, the bioconductor “limma” package^14^ was used for background correction, data normalisation (using the “neqc” function)^31^ and DGE analysis^32^ for paired samples (using the “treat” function) in the 18 subjects across all 8 time points (i.e. Base1, Base2, EPO3, EPO4, EPO5, Post6, Post7 and Post8). Notably, only probes expressed in at least 7 samples at a detection *p* < 0.05 were kept. Probes were annotated to illuminaHumanv4.db^33^ and only probes with “good” and “perfect” matching quality were retained followed by removing probes with “NA” or multiple mappings. Probes with low expression values below 5.1 were excluded prior to the DGE analysis (assessed using the limma “plotSA” function). Transcripts were considered significantly expressed for a fold change of 1.2 bounded to a 5% false discovery rate (FDR) (thereby, giving more weight to fold change for gene ranking). These are common cut-off values being used for declaring biologically and statistically significant findings in a DGE analysis^34^.

### Microarray analysis with the GeneChip HTA2.0

One hundred nanograms of total RNA was processed using the GeneChip WT Plus Reagent Kit according to the manufacturer’s instructions (Thermo Fisher Scientific, Waltham, MA, US) for 10 out of the 18 subjects at the selected time points (i.e. Base1, Base2, EPO3, EPO4 and Post7). Single-stranded cDNA (ss-cDNA) was synthesised by the reverse transcription of cRNA. Two hundred microlitres of hybridisation cocktail (containing approximately 5.2 μg fragmented and labelled ss-cDNA) was loaded onto the GeneChip HTA2.0 (Thermo Fisher Scientific, Waltham, MA, US). The GeneChip arrays were incubated in the GeneChip Hybridization Oven 645 for 16 h, washed and stained on the GeneChip Fluidics Station 450. The arrays were then scanned using the GeneChip Scanner 3000 7G. The Applied Biosystems Transcriptome Analysis Console (version:4.0.1.36; Thermo Fisher Scientific, Waltham, MA, US) was used to perform initial data QC and data visualisation. The relative log expression box plots were plotted following the quality assessment steps illustrated in Ref^35^. The Bioconductor “oligo” package^36^ was used to read in the raw intensity CEL files, and the “rma” function was used for background correction, normalisation, and data summarisation to the gene level (defined by the argument “core”). Probes were annotated to hta20transcriptcluster.db^37^ and probes with “NA” or multiple mappings were removed. The “limma” package was then used to perform the usual DGE analysis for paired samples (the analysis setting is identical to that used in the Illumina microarray analysis illustrated above). Transcript clusters (loosely equal to genes) were considered significantly expressed at a fold change of 1.2 bounded to a 5% FDR.

### RNA-seq on the illumina NextSeq 500

Five hundred nanograms of total RNA was used for sequencing according to the Illumina TruSeq Stranded mRNA sample prep guide—high sample protocol (Illumina, San Diego, CA, USA). Briefly, mRNA molecules were purified using the poly-T oligo attached magnetic beads following which the mRNA was fragmented and primed for cDNA synthesis. A single “A” base was subsequently added to the 3-prime end of the synthesised blunt-ended cDNA and ligated with index adapters for hybridisation onto a flow cell. The DNA fragments with adapters on both ends were amplified via polymerase chain reaction to generate the final double-stranded cDNA (ds-cDNA) library followed by library validation and normalisation and pooling of the samples. Samples were pooled and then sequenced at 2 × 75 bp read length to a depth aimed at approximately 64 M reads per sample on the Illumina NextSeq 500 (Illumina, San Diego, CA, USA). Ten out of the 18 subjects at the selected time points (i.e. Base1, Base2, EPO3, EPO4, and Post7) were analysed. Raw sequences were examined by FastQC^38^ for basic quality checks (e.g. per base sequence quality, adaptor content, and per base N content), FastQ Screen^39^ for mapping against multiple reference genomes for detecting sample swaps or sample contamination that may have resulted from sources other than humans (i.e. in this case, mapping against human, mouse and rat genomes were conducted), and HISAT2^9^ for alignment to the reference genome assembly (GRCh38.p12^10^) using the Ensembl 94 annotation^40^ prior to RseQC^11^ for read distribution analysis. Salmon^12^ was used for aligning to the Ensembl transcriptome fasta file “Homo_sapiens.GRCh38.cdna.all.fa.gz” for transcripts quantification (using selective alignment with the *decoy aware* target transcriptome to eliminate potential spurious mapping to unannotated genomic locus over a *k*-mer length of 31, along with –SeqBias and –gcBias flags switched on to correct for any unwanted effects), followed by bioconductor package “tximport”^41^ for summarising transcript-level estimates to genes based on the Ensembl release 94^40^, and DESeq2^13^ for paired sample DGE analysis. Pre-filtering was performed to keep genes that have at least 5 reads in 4 samples prior to the DGE analysis. Ensembl IDs were mapped to gene symbols using the bioconductor package “org.Hs.eg.db”^42^ and un-defined mappings were removed (i.e. gene with “NA” or multiple mappings). MultiQC^43^ was used to aggregate the analysis results from the FastQC, FastQ Screen and RseQC runs from multiple samples. PCA for the top 500 genes of high variance and gene clustering analysis for the top 30 genes were performed following the DESeq2 vignette on data quality assessment procedures^44^. The bioconductor package “SVA”^45^ was used to assess any surrogate variables that may represent other variations in the data for further correction. Shrinkage estimator “apeglm” was used for the shrinkage of log fold change estimates and for ranking genes by effect size^46^. Genes exceeding a fold change of 1.2 bounded to the default *s *value < 0.005 were reported.

### RNA-seq on the MGI DNBSEQ-G400RS

Four hundred nanograms of total RNA was used for sequencing on the MGI DNBSEQ-G400RS instrument (MGI, Shenzhen, China). Total RNA was first treated with Globin-Zero Gold Kit (Illumina, San Diego, CA, USA) for rRNA depletion and globin mRNA reduction. The ds-cDNA library preparation is in line with the Illumina RNA-seq protocol described in the above section. The ds-cDNAs were then heat denatured and circularised by the splint oligo sequence to generate the single strand circle DNA followed by rolling circle replication to create DNA nanoballs (DNB) for processing on the MGI DNBSEQ-G400RS. The same 50 samples used for the GeneChip and Illumina RNA-seq profiling were again analysed on this platform. These samples were sequenced on 6 flowcells at 2 × 100 bp read length aimed at a sequencing depth of 64 M reads. Raw sequences were processed for quality assessment, alignment, transcripts quantification and DGE analysis, in line with the process described in the “RNA-seq on the illumina NextSeq 500” section above. The same cut-offs as illustrated in the Illumina RNA-seq section for defining a significant result were applied (i.e. a fold change of 1.2 bounded to the default *s *value < 0.005).

### Gene set enrichment analyses in GSEA and reactome

The pathway enrichment analysis was performed in accordance with recommendations from Ref^47^, where appropriate. Specifically, normalised RNA-seq counts (outputted from DESeq2 “counts” function with the argument “normalized = TRUE”) and normalised microarray gene expression values were subjected to gene set enrichment analysis using GSEA (v4.0.3)^15,16^ by examining the Molecular Signatures Database (MSigDB)^15,17^ Hallmark (H; containing 50 gene sets)^18^ and Gene Ontology (C5; BP: subset of GO biological processes containing 7,573 gene sets)^19,20^ collections of functional gene sets. Low count genes (by removing genes with counts below 5 in at least 4 samples) and genes with unidentified mappings from RNA-seq, as well as control probes, low-quality probes and probes with unidentified mappings from microarray analyses were excluded from the expression datasets prior to the GSEA. A standard GSEA run was applied for each dataset by performing 1000 phenotype permutations and by collapsing the Ensembl IDs and probe IDs to gene symbols by mapping to their corresponding chip platforms available from the MSigDB database (i.e. Human_ENSEMBL_Gene_ID_MSigDB.v7.2.chip for RNA-seq, Human_AFFY_hta_2_0_MSigDB.v7.2.chip for GeneChip HTA2.0 and Human_Illumina_HumanHT_12_v4_Array_MSigDB.v7.2.chip for Illumina BeadChip). Other main parameters used in a GSEA run included the default ranking metric “Signal2Noise”, gene set size filters (15–200 for H, and 10–500 for C5) and collapsing mode (“Sum_of_probes” for RNA-seq, and “Max_probe” for microarray). Default values were used for other fields of the GSEA run. EnrichmentMap App^48^ was used for creating biological networks of the GSEA pathways (pathway FDR < 0.1, nominal P < 0.05 and Jaccard Overlap coefficient > 0.375 with combined constant k = 0.5) and AutoAnnotate App^49^ for gene sets annotation and clustering (MCL Cluster annotation) in Cytoscape (v3.8.0)^50^. The most significantly enriched gene set was used to label a gene set cluster, characterised by the NES.

Raw counts from the RNA-seq (outputted from DESeq2 “counts” function by setting “normalized = FALSE”), and normalised and log2 transformed gene expression values from microarray analyses were uploaded onto Reactome (v73)^24^ for quantitative pathway analysis (ReactomeGSA) using the PADOG algorithm^51,52^ for gene expression visualisation in pathway diagrams. Protein–protein interactors derived from the IntAct database^53^ with the IntAct score ≥ 0.556 (of medium to high confidence interactions) were included in the analysis to improve the Reactome pathway coverage. For consistency, these expression datasets were collapsed to gene symbols (described above) using the “Collapse Dataset” tool in the GSEA software prior to the ReactomeGSA.

### Cross-platform comparison and data triangulation

Direct comparisons for the coding gene features identified across the MGI DNBSEQ-G400RS, Illumina NextSeq 500 and GeneChip HTA2.0 platforms in the 10 subjects (comprised of 50 samples) were carried out on the differentially expressed genes following the DESeq2/limma DGE analyses. A sankey diagram was plotted for visualisation of the DGE results using the “ggalluvial” package^54^. The cross-platform correlations were computed using the “ggscatter” function in the package “ggpubr”^55^. “ggplot2”^56^ and “cowplot”^57^ packages were used for creating publication-quality figures. Leading edge genes from the significantly expressed GSEA pathways (derived from MGI DNBSEQ-G400RS, GeneChip HTA2.0 and HumanHT-12 v4.0 Expression BeadChip; including all pathways with the NES > 1.9 or the representative pathways of gene set clusters when the NES < 1.9) were extracted and compared to the DGE genes to generate the common sets of genes identified by both the GSEA and DGE analyses. These genes were then overlapped with the DGE results obtained from the Illumina NextSeq 500 for confirmation. The interaction networks among pathway genes were determined by expression overlay with the Reactome pathway diagrams, focusing on the networks represented by the confirmed genes above (originated from the MGI RNA-seq and BeadChip datasets). The final lists of genes were obtained by overlapping the significantly altered genes and their interactors involved in these Reactome networks with the DGE genes derived from the DESeq2/limma analyses. Venn Diagrams illustrating the genes were plotted using an online version of the diagram tool, draw.io^58^.

## Supplementary Information

Supplementary Information 1.

Supplementary Information 2.

## Data Availability

RNA-seq raw sequence data and read counts are available at the NCBI Gene Expression Omnibus under accession no. GSE186294. Raw and processed microarray data are available at ArrayExpress under accession no. E-MTAB-11080 (GeneChip HTA2.0) and E-MTAB-2874 (HT12v4 BeadChip; previously deposited). All other data are present either in the main text or the supplementary information.
