# Supplementary material for: Cross-platform transcriptomic profiling of the response to recombinant human erythropoietin
Source: Sci Rep. 2021 Nov 4;11:21705. doi: 10.1038/s41598-021-00608-9 (PMC8568984; doi:10.1038/s41598-021-00608-9)
Supplement: Supplementary file 1 — Supplementary Information 1. [file 41598_2021_608_MOESM1_ESM.zip › Supplementary Data 1-16/Supplementary Data 11/Notes.docx]

For the following Reactome pathways (snapshots), the authors of the original pathway diagrams are included below and should be credited:

**R-HSA-917937:** Stephan R, D’Eustachio P.

**R-HSA-162909:** Benarous, Zhao RY, Peterlin BM.

**R-HSA-5688426:** Jupe S, Meldal BH.

**R-HSA-425366:** Jassal B, He L.

The authors are affiliated to European Bioinformatics Institute; New York University Langone Medical Center; Ontario Institute for Cancer Research; Oregon Health and Science University.
