# Supplementary material for: Cross-platform transcriptomic profiling of the response to recombinant human erythropoietin
Source: Sci Rep. 2021 Nov 4;11:21705. doi: 10.1038/s41598-021-00608-9 (PMC8568984; doi:10.1038/s41598-021-00608-9)
Supplement: Supplementary file 1 — Supplementary Information 1. [file 41598_2021_608_MOESM1_ESM.zip › Supplementary Data 1-16/Supplementary Data 11/R-HSA-977225-SNCA-P8-Beadchip.pdf]

Amyloid fiber formation

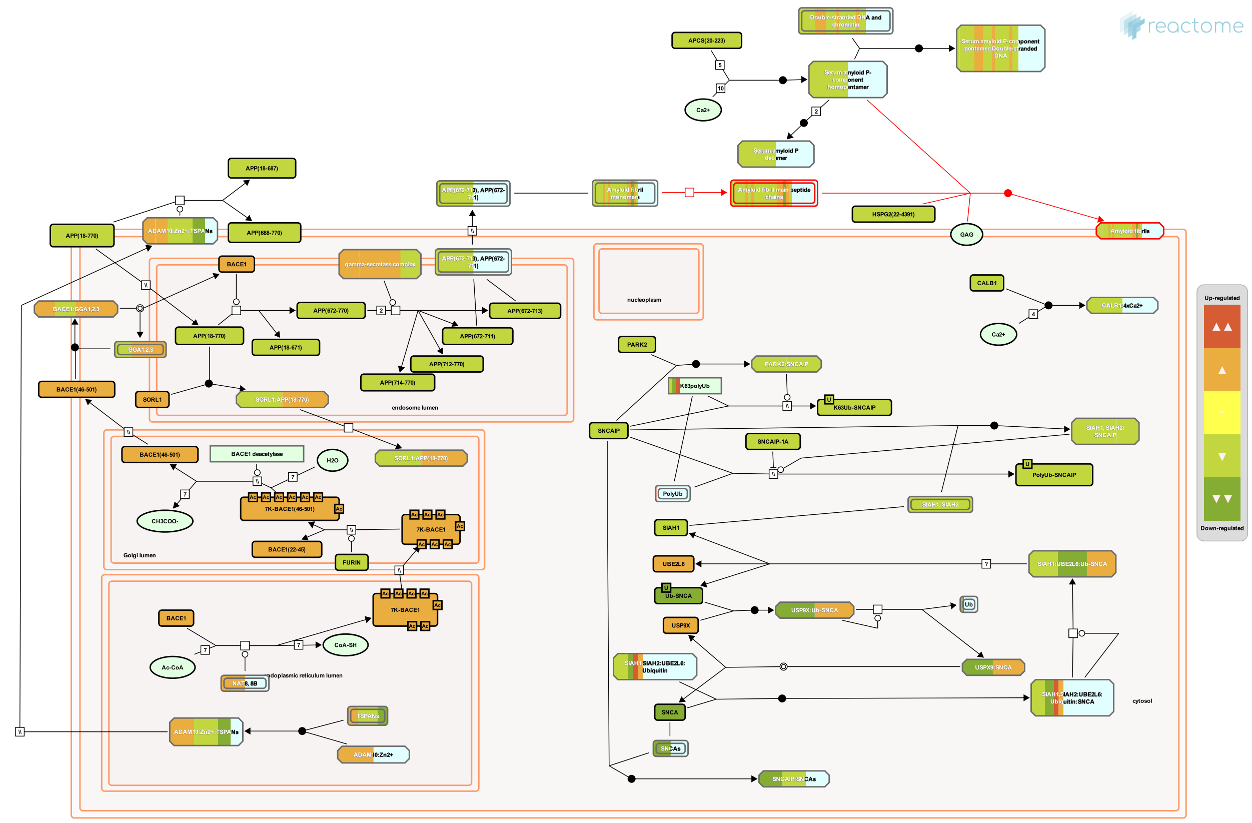

D'Eustachio, P., Jassal, B., Jupe, S., Meras-Rios, A., Perry, G.

European Bioinformatics Institute, New York University Langone Medical Center, Ontario Institute for Cancer Research, Oregon Health and Science University.

The contents of this document may be freely copied and distributed in any media, provided the authors, plus the institutions, are credited, as stated under the terms of Creative Commons Attribution 4.0 International (CC BY 4.0) License. For more information see our license.

28/09/2020

Introduction

Reactome is open-source, open access, manually curated and peer-reviewed pathway database. Pathway annotations are authored by expert biologists, in collaboration with Reactome editorial staff and cross-referenced to many bioinformatics databases. A system of evidence tracking ensures that all assertions are backed up by the primary literature. Reactome is used by clinicians, geneticists, genomics researchers, and molecular biologists to interpret the results of high-throughput experimental studies, by bioinformaticians seeking to develop novel algorithms for mining knowledge from genomic studies, and by systems biologists building predictive models of normal and disease variant pathways.

The development of Reactome is supported by grants from the US National Institutes of Health (P41 HG003751), University of Toronto (CFREF Medicine by Design), European Union (EU STRP, EMI-CD), and the European Molecular Biology Laboratory (EBI Industry program).

Literature references

Fabregat, A., Sidiropoulos, K., Viteri, G., Forner, O., Marin-Garcia, P., Arnau, V. et al. (2017). Reactome pathway analysis: a high-performance in-memory approach. *BMC bioinformatics, 18*, 142. ↗

Sidiropoulos, K., Viteri, G., Sevilla, C., Jupe, S., Webber, M., Orlic-Milacic, M. et al. (2017). Reactome enhanced pathway visualization. *Bioinformatics, 33*, 3461-3467. ↗

Fabregat, A., Jupe, S., Matthews, L., Sidiropoulos, K., Gillespie, M., Garapati, P. et al. (2018). The Reactome Pathway Knowledgebase. *Nucleic Acids Res, 46*, D649-D655. ↗

Fabregat, A., Korninger, F., Viteri, G., Sidiropoulos, K., Marin-Garcia, P., Ping, P. et al. (2018). Reactome graph database: Efficient access to complex pathway data. *PLoS computational biology, 14*, e1005968. ↗

Reactome database release: 73

This document contains 1 pathway and 33 reactions (see Table of Contents)

Analysis properties
