# Supplementary figures and images for: Cross-platform transcriptomic profiling of the response to recombinant human erythropoietin
Source: Sci Rep. 2021 Nov 4;11:21705. doi: 10.1038/s41598-021-00608-9 (PMC8568984; doi:10.1038/s41598-021-00608-9)

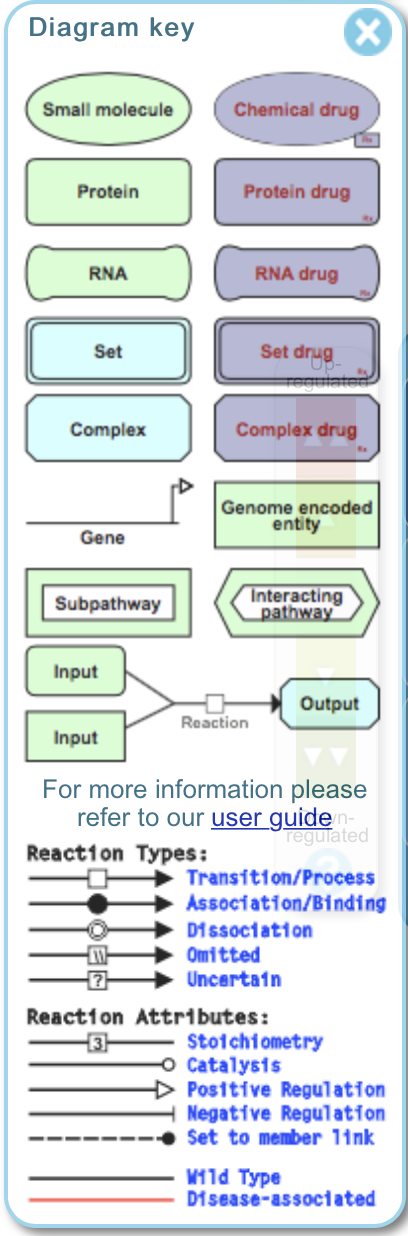

Supplement: Supplementary file 1 — Supplementary Information 1. [file 41598_2021_608_MOESM1_ESM.zip › Supplementary Data 1-16/Supplementary Data 11/Diagram key.png]

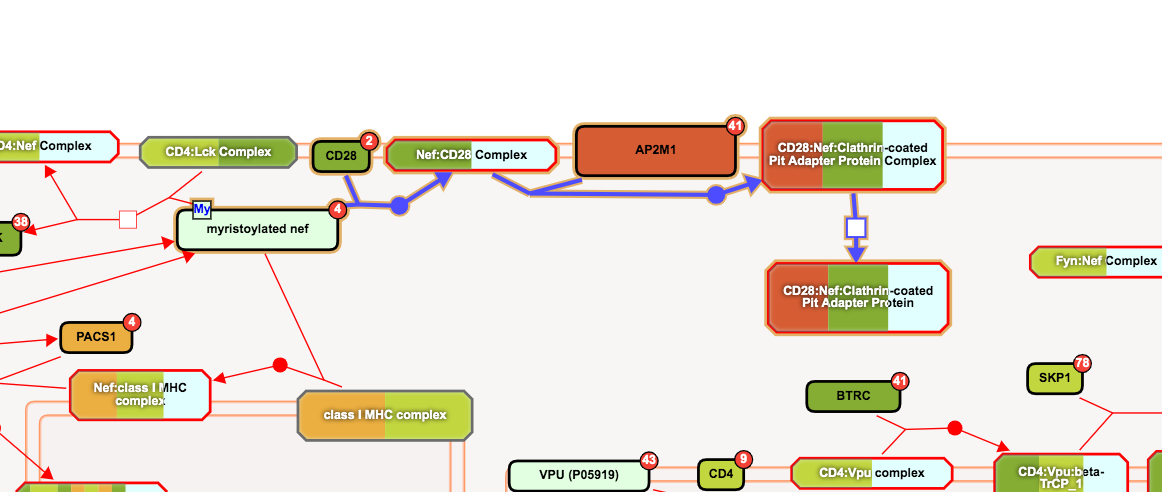

Supplement: Supplementary file 1 — Supplementary Information 1. [file 41598_2021_608_MOESM1_ESM.zip › Supplementary Data 1-16/Supplementary Data 11/R-HSA-162909-MPP1-E4:5-Beadchip.png]

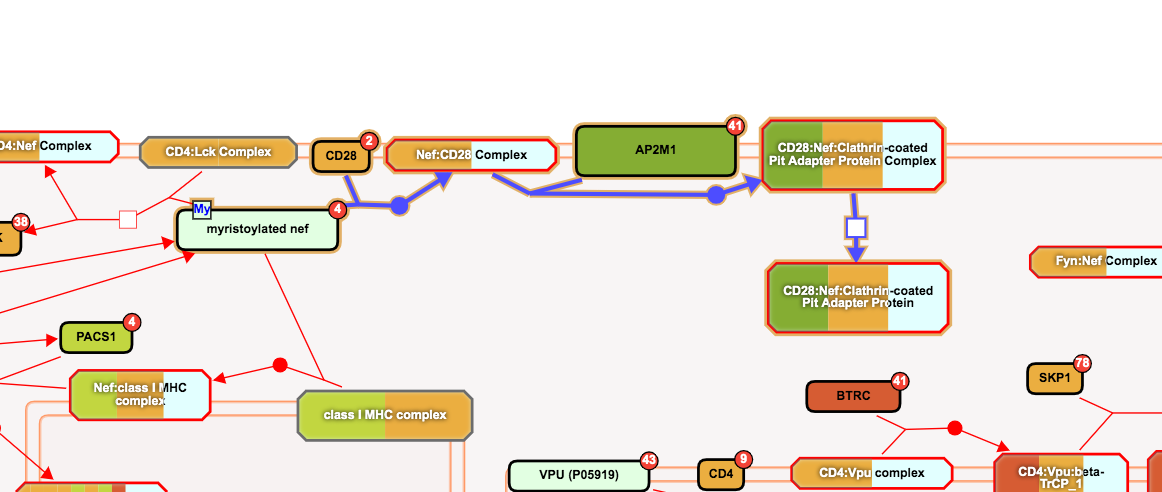

Supplement: Supplementary file 1 — Supplementary Information 1. [file 41598_2021_608_MOESM1_ESM.zip › Supplementary Data 1-16/Supplementary Data 11/R-HSA-162909-MPP1-P7-Beadchip.png]

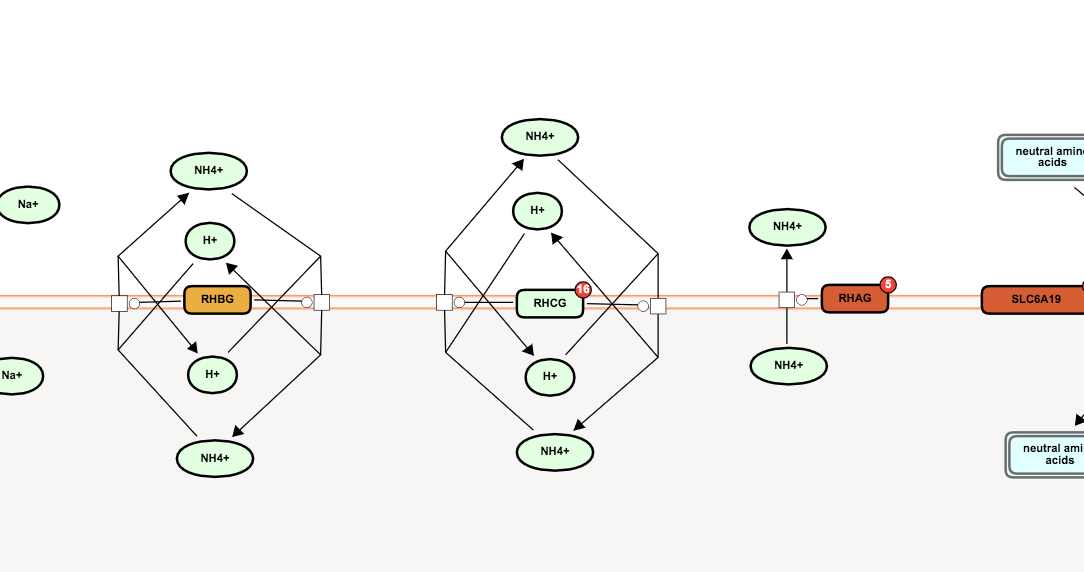

Supplement: Supplementary file 1 — Supplementary Information 1. [file 41598_2021_608_MOESM1_ESM.zip › Supplementary Data 1-16/Supplementary Data 11/R-HSA-425366-RHAG-E4-MGI-Rhesus glycoproteins mediate ammonium transport.png]

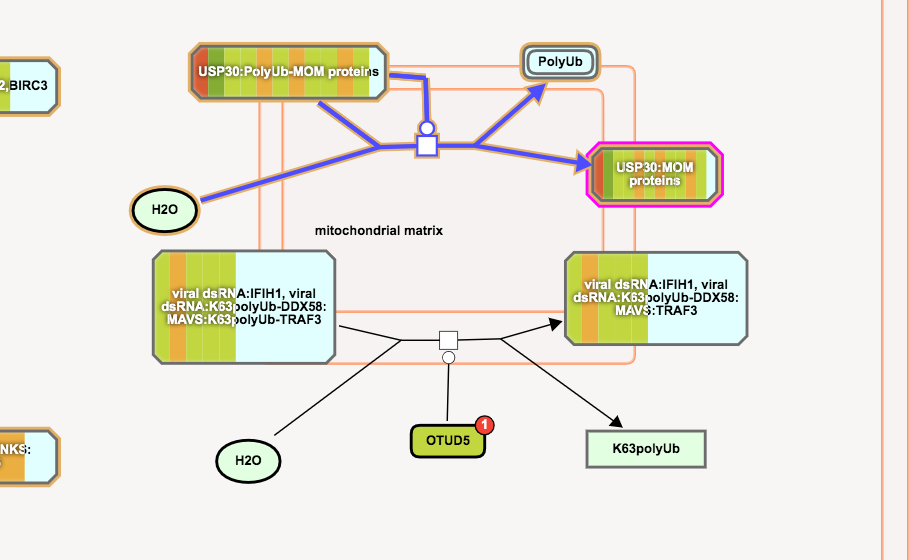

Supplement: Supplementary file 1 — Supplementary Information 1. [file 41598_2021_608_MOESM1_ESM.zip › Supplementary Data 1-16/Supplementary Data 11/R-HSA-5688426-FKBP8-E5-Beadchip.png]

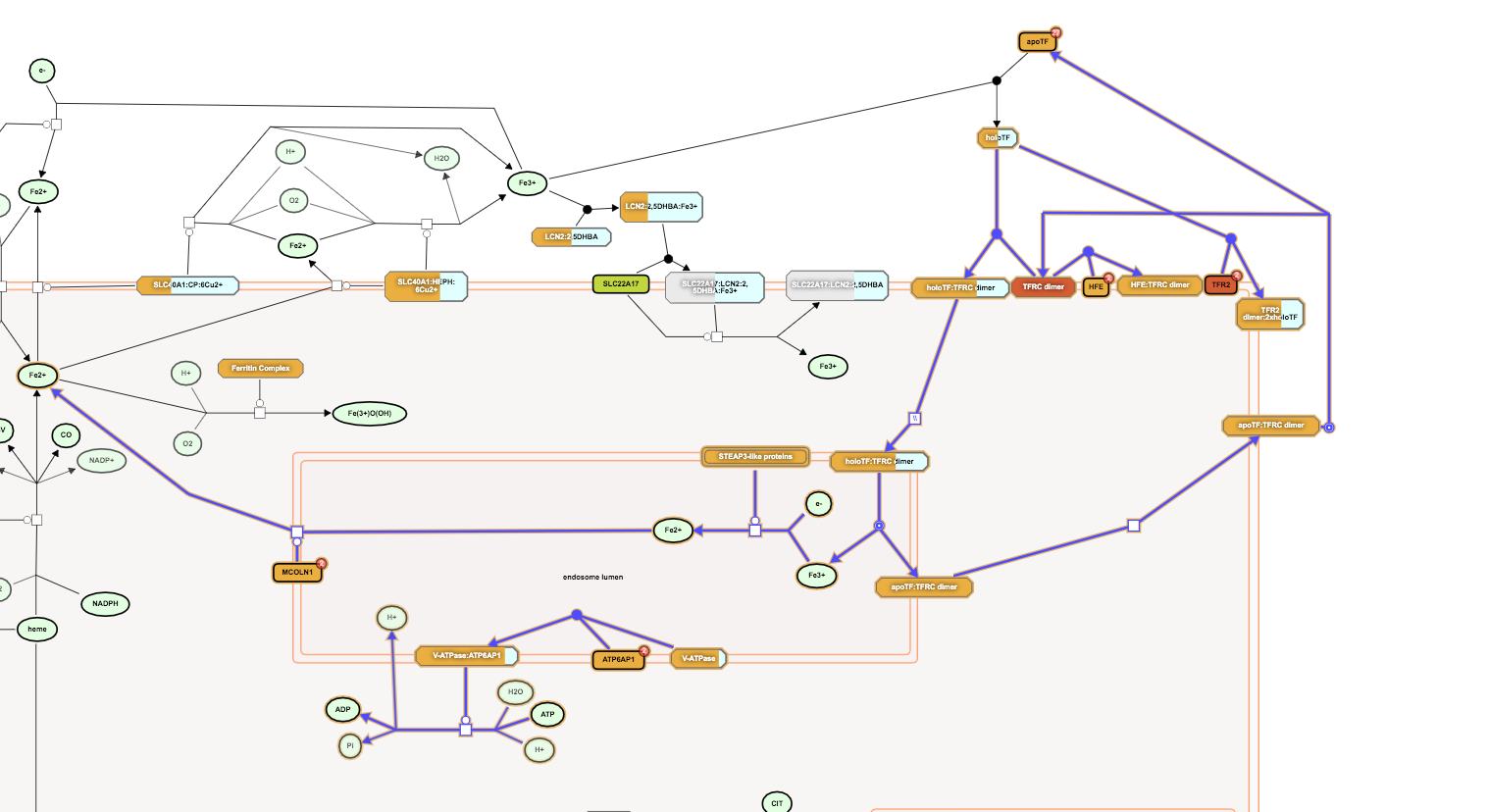

Supplement: Supplementary file 1 — Supplementary Information 1. [file 41598_2021_608_MOESM1_ESM.zip › Supplementary Data 1-16/Supplementary Data 11/R-HSA-917937-FAM210B-E4-MGI.png]
