# Supplementary material for: Cross-platform transcriptomic profiling of the response to recombinant human erythropoietin
Source: Sci Rep. 2021 Nov 4;11:21705. doi: 10.1038/s41598-021-00608-9 (PMC8568984; doi:10.1038/s41598-021-00608-9)
Supplement: Supplementary file 2 — Supplementary Information 2. [file 41598_2021_608_MOESM2_ESM.pdf]

Supplementary Information for

Cross-platform transcriptomic profiling of the response to recombinant human erythropoietin

Guan Wang[1,2]*, Traci Kitaoka[3], Ali Crawford[3], Qian Mao[4], Andrew Hesketh[5], Fergus M. Guppy[5,6], Garrett I. Ash[7,8], Jason Liu[9], Mark B. Gerstein[9-12], Yannis P. Pitsiladis[6]*

*Corresponding authors. Email: g.wang2@brighton.ac.uk; y.pitsiladis@brighton.ac.uk.

This document includes:

Supplementary Figs. S1 to S10
Supplementary Table S1 to S7
Captions for Supplementary Data 1 to 16

Other Supplementary Information for this manuscript include the following:

Supplementary Data 1 to 16

FastQC: Mean Quality Scores

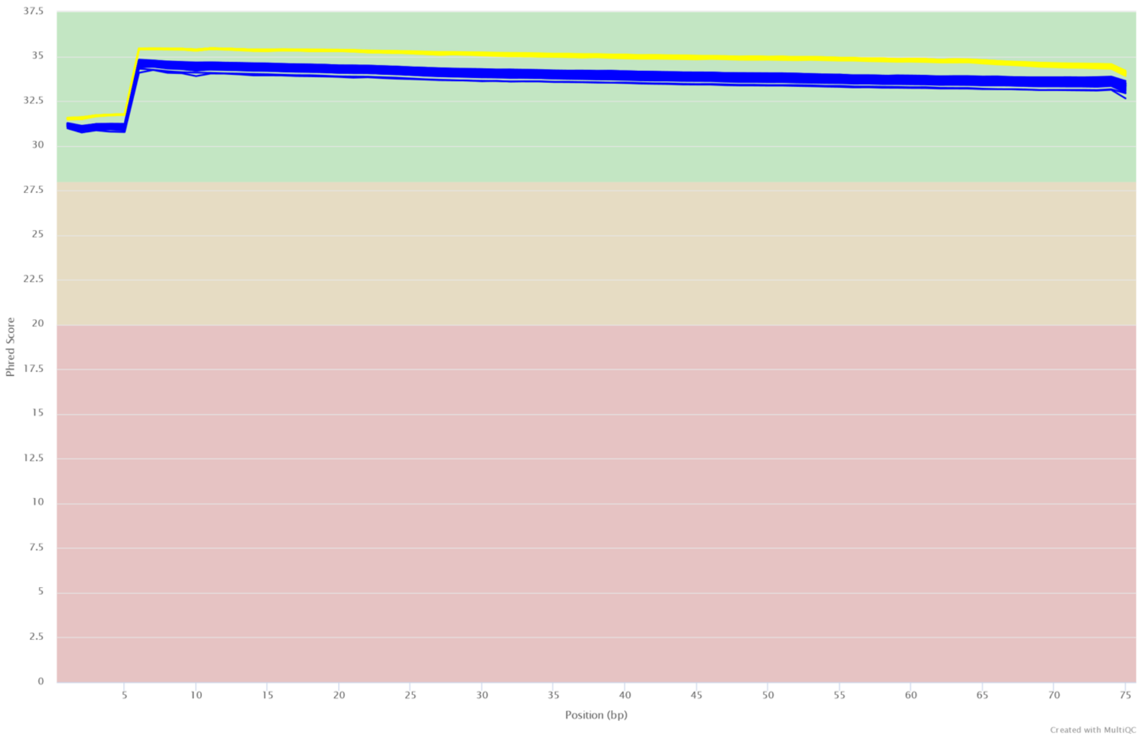

FastQC: Mean Quality Scores

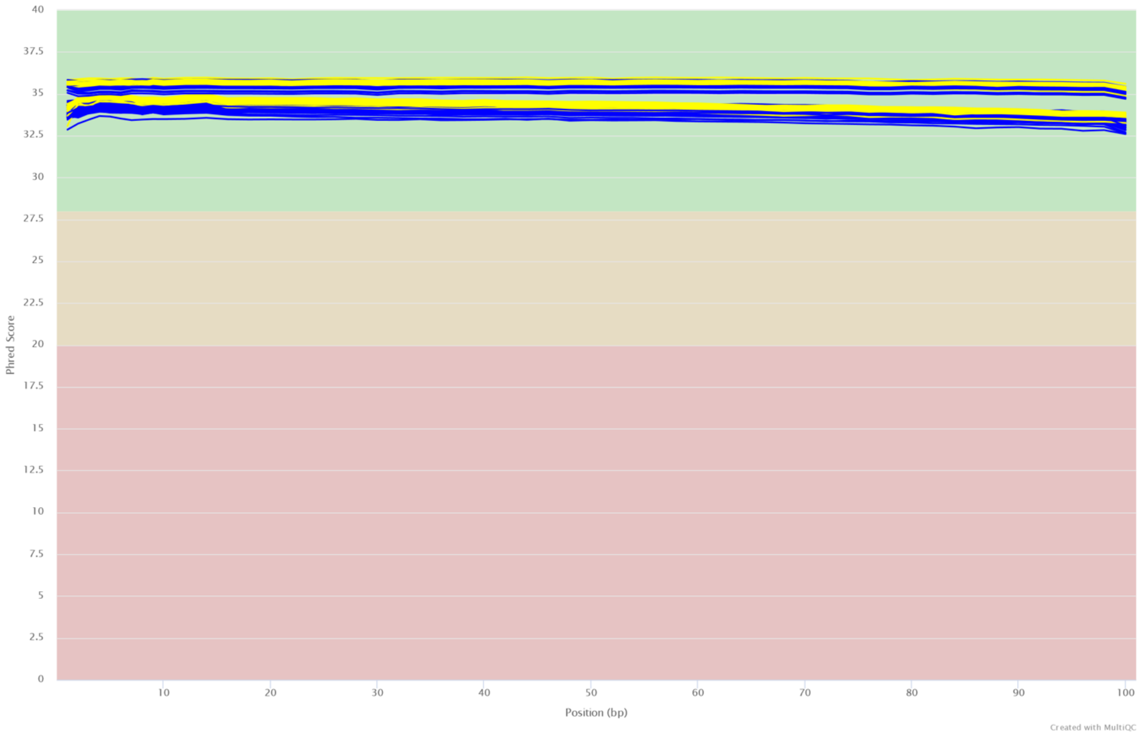

Supplementary Fig. S1. FastQC per base sequence quality scores of the Illumina (upper panel; excluding sample 33 and 40) and MGI (lower panel) sequencing reads. Yellow Line: Read 1 data; Blue Line: Read 2 data.

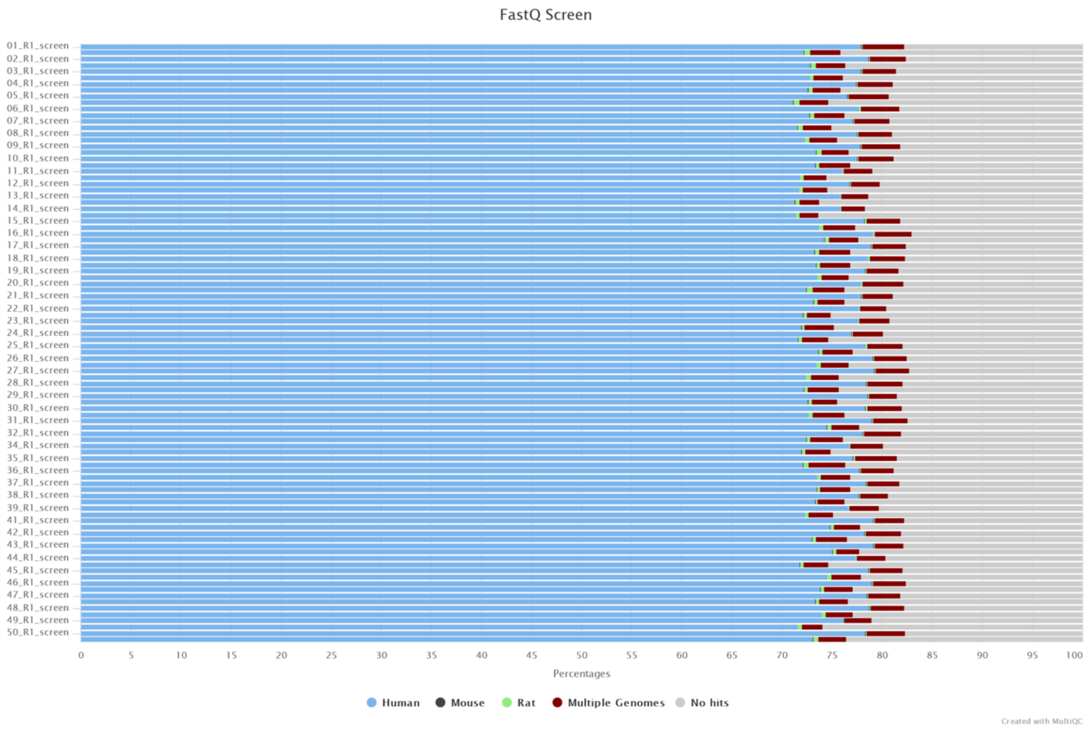

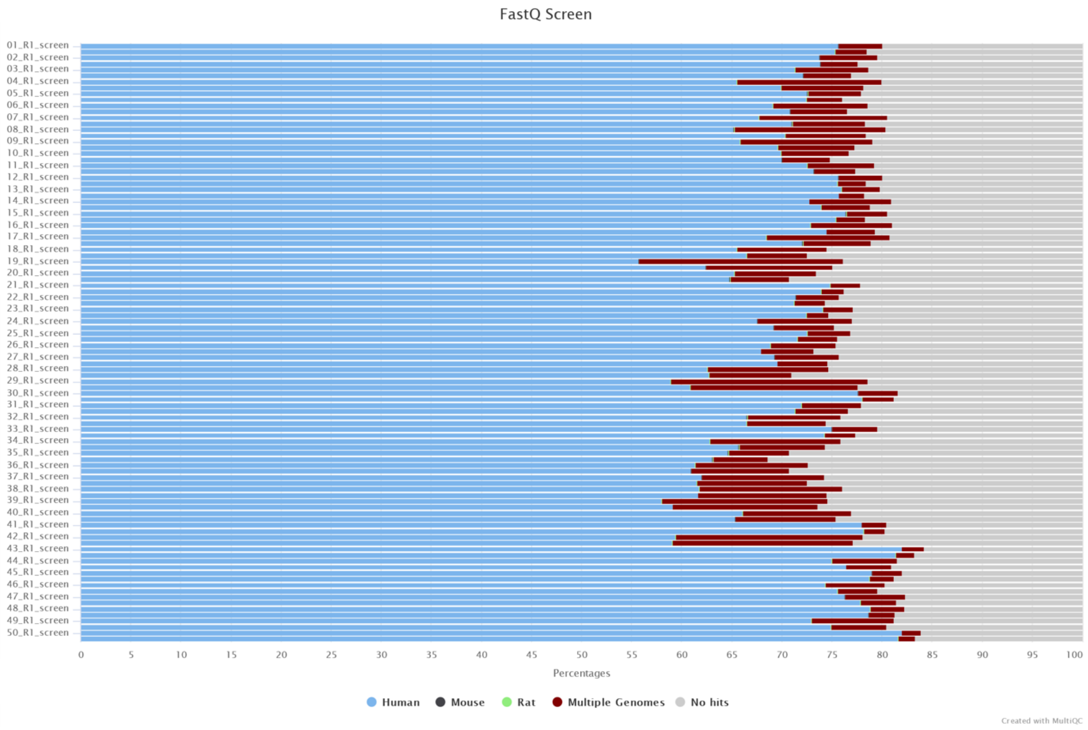

Supplementary Fig. S2. FastQ Screen results plotted against human, mouse and rat genomes for Read 1 (R1) and Read 2 (R2) sequences showing the library compositions to detecting potential sample contamination following RNA-seq on the Illumina (upper panel; excluding sample 33 and 40) and MGI (lower panel) platforms.

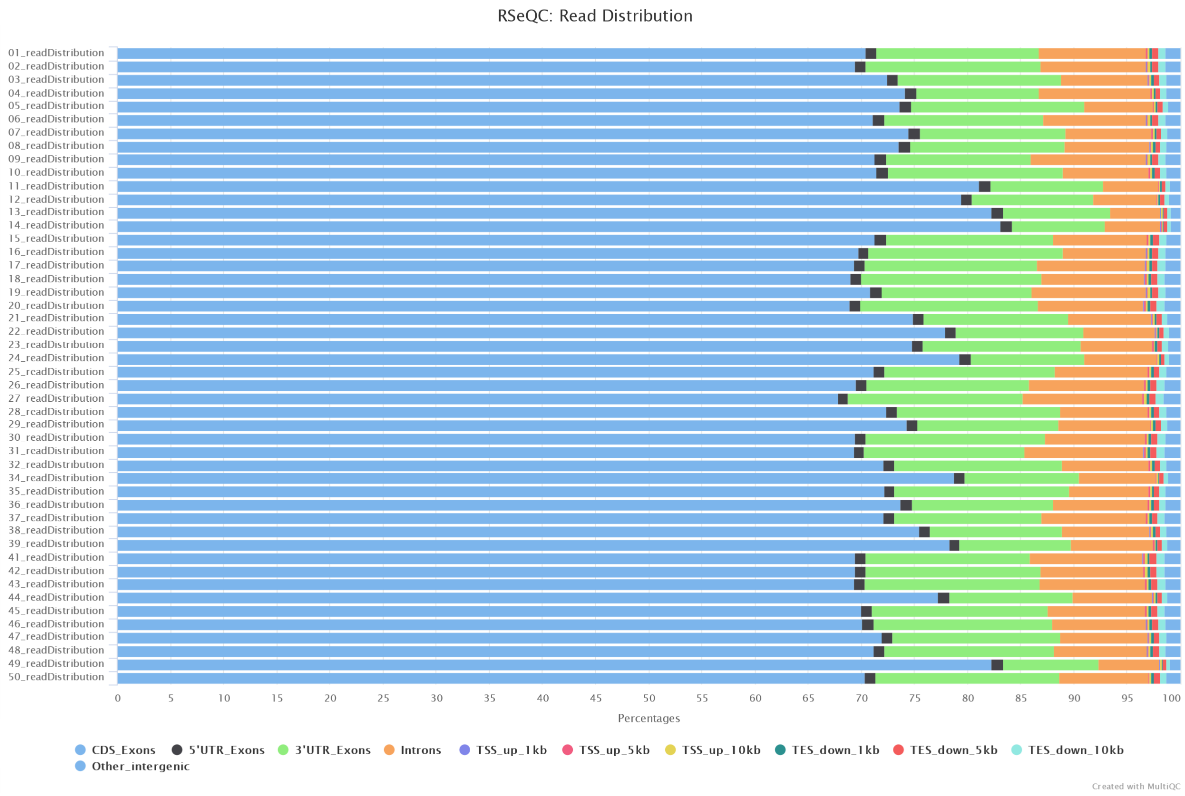

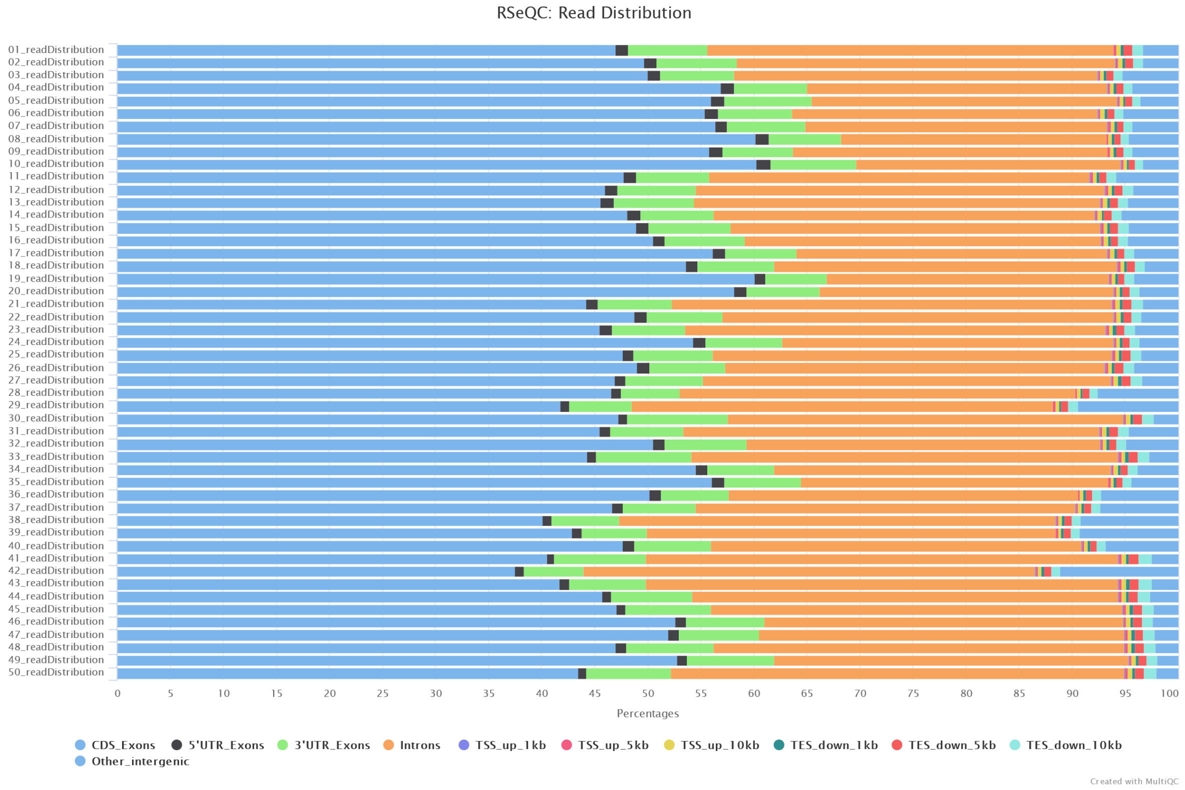

Supplementary Fig. S3. RSeQC read distribution across the Illumina (N=48; upper panel) and MGI (N=50; lower panel) sequencing platforms.

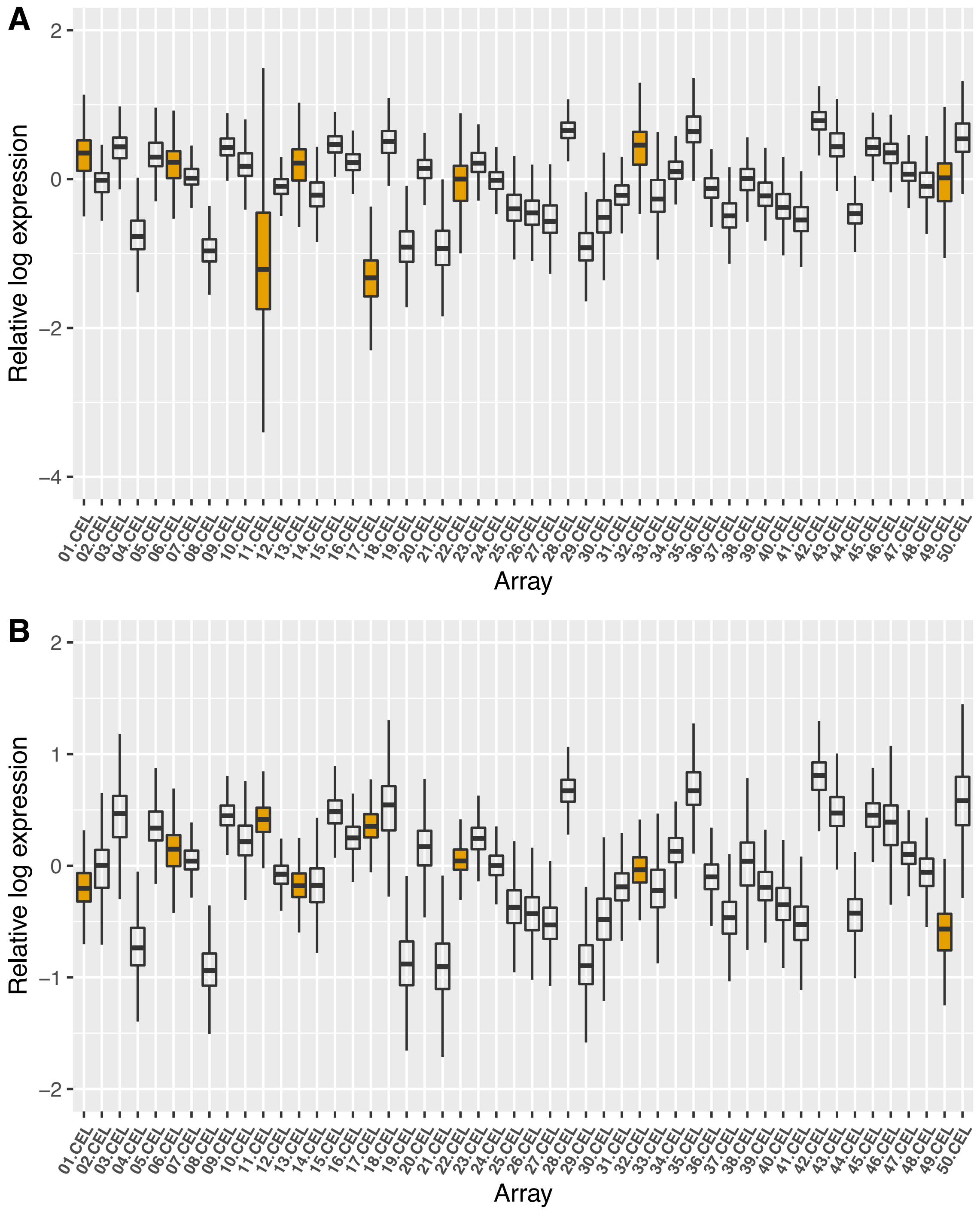

Supplementary Fig. S4. Distribution of the relative log_2 expression of background corrected intensities of each transcript against their median intensities across all samples tested on the GeneChip arrays. Panel A shows eight samples (boxes filled in yellow) with relatively high variability compared to other samples in the dataset. Panel B illustrates the improved intensity distribution of the eight samples after target re-prep and re-scanning.

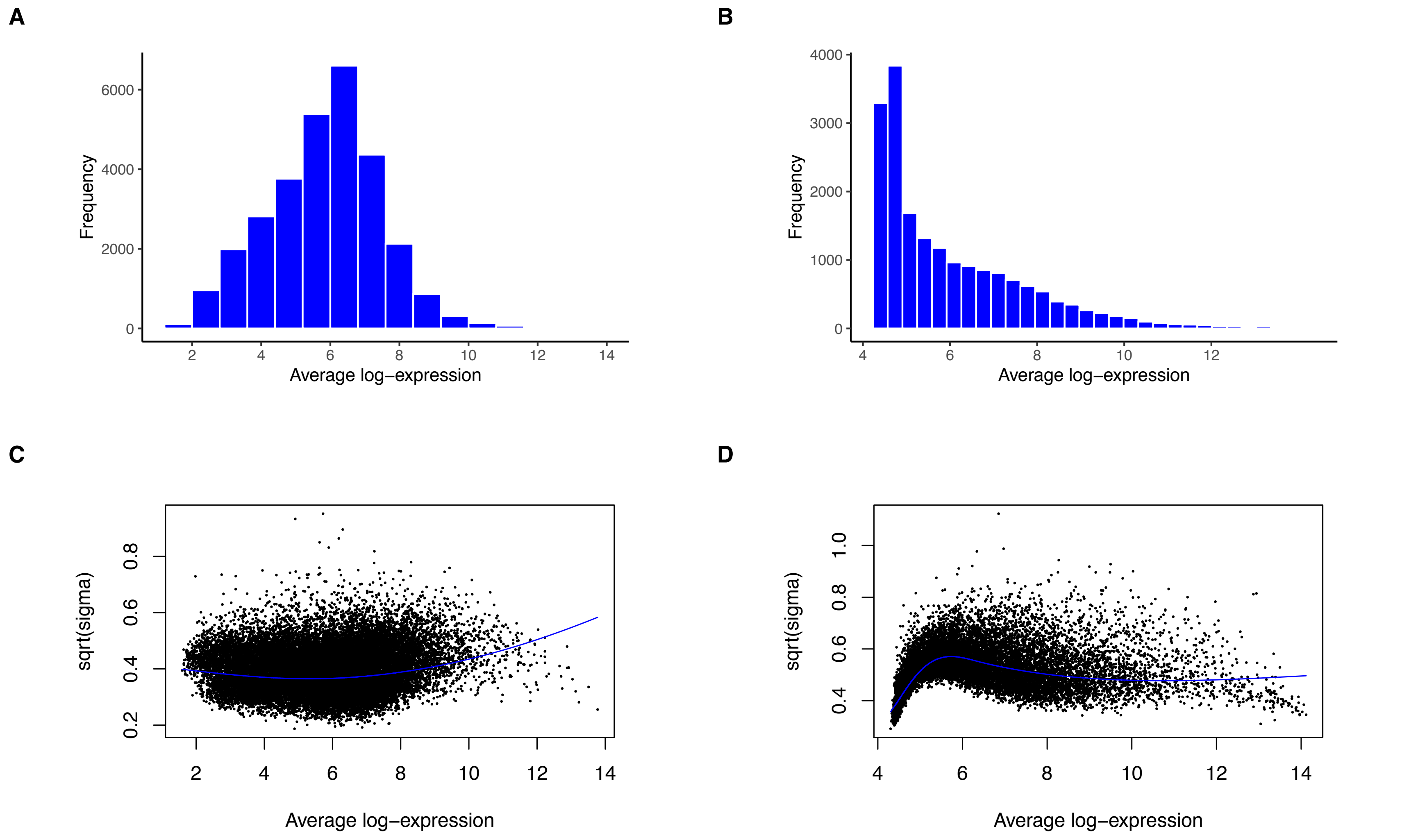

Supplementary Fig. S5. Histograms of the average $\log_2$ expression and the mean-variance limma-trend plots of the detected features on the GeneChip (panel A and C) and BeadChip (panel B and D) platforms. sigma: the estimated residual standard deviation. Blue line: the fitted limma trend in C and D.

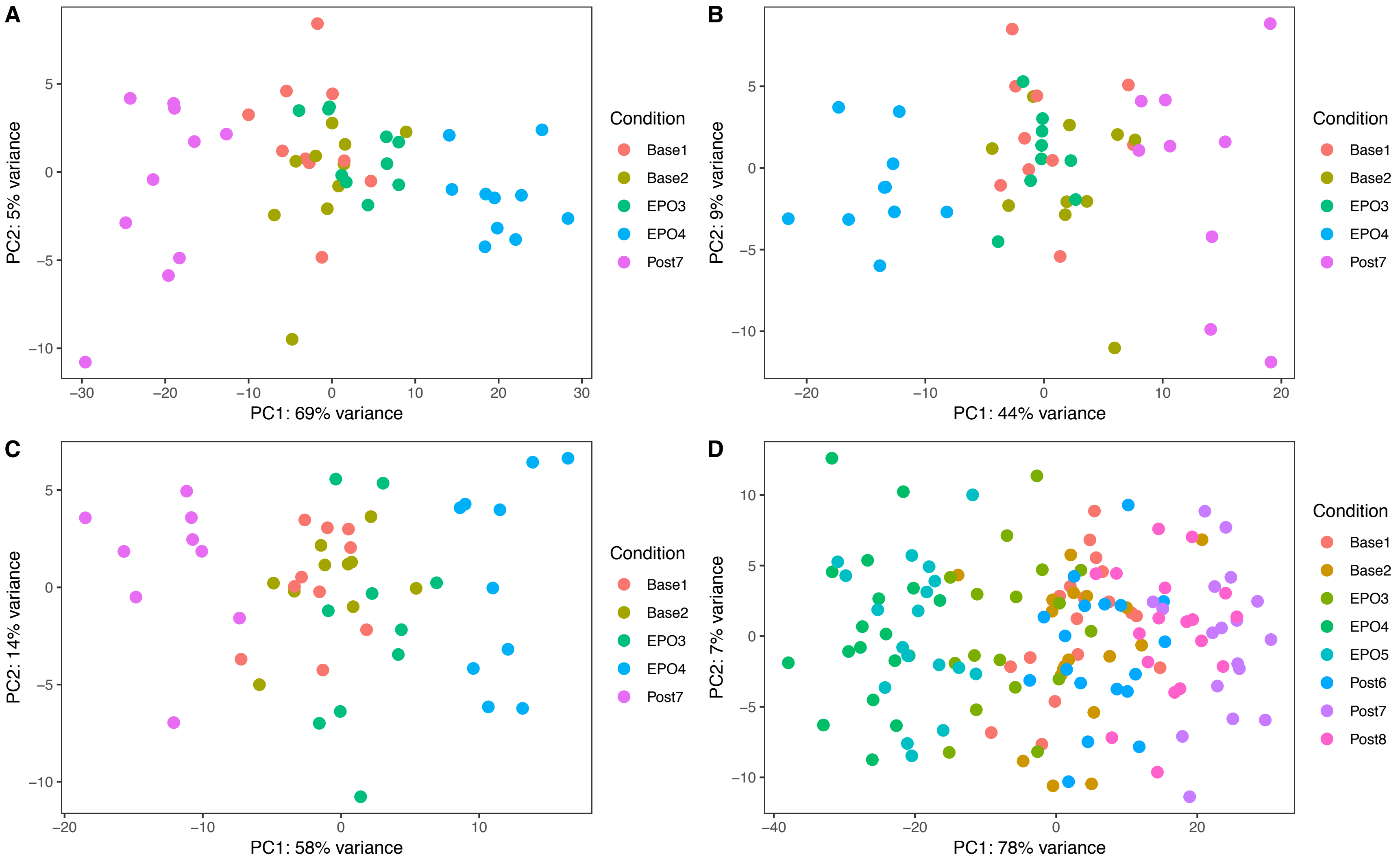

Supplementary Fig. S6. Principal component analysis of the top 500 genes ranked by the sample variance across the RNA-seq and microarray platforms. A: MGI DNBSEQ-G400RS, B: Illumina NextSeq 500; C: GeneChip HTA2.0; D: Illumina HumanHT-12 v4 Expression BeadChip.

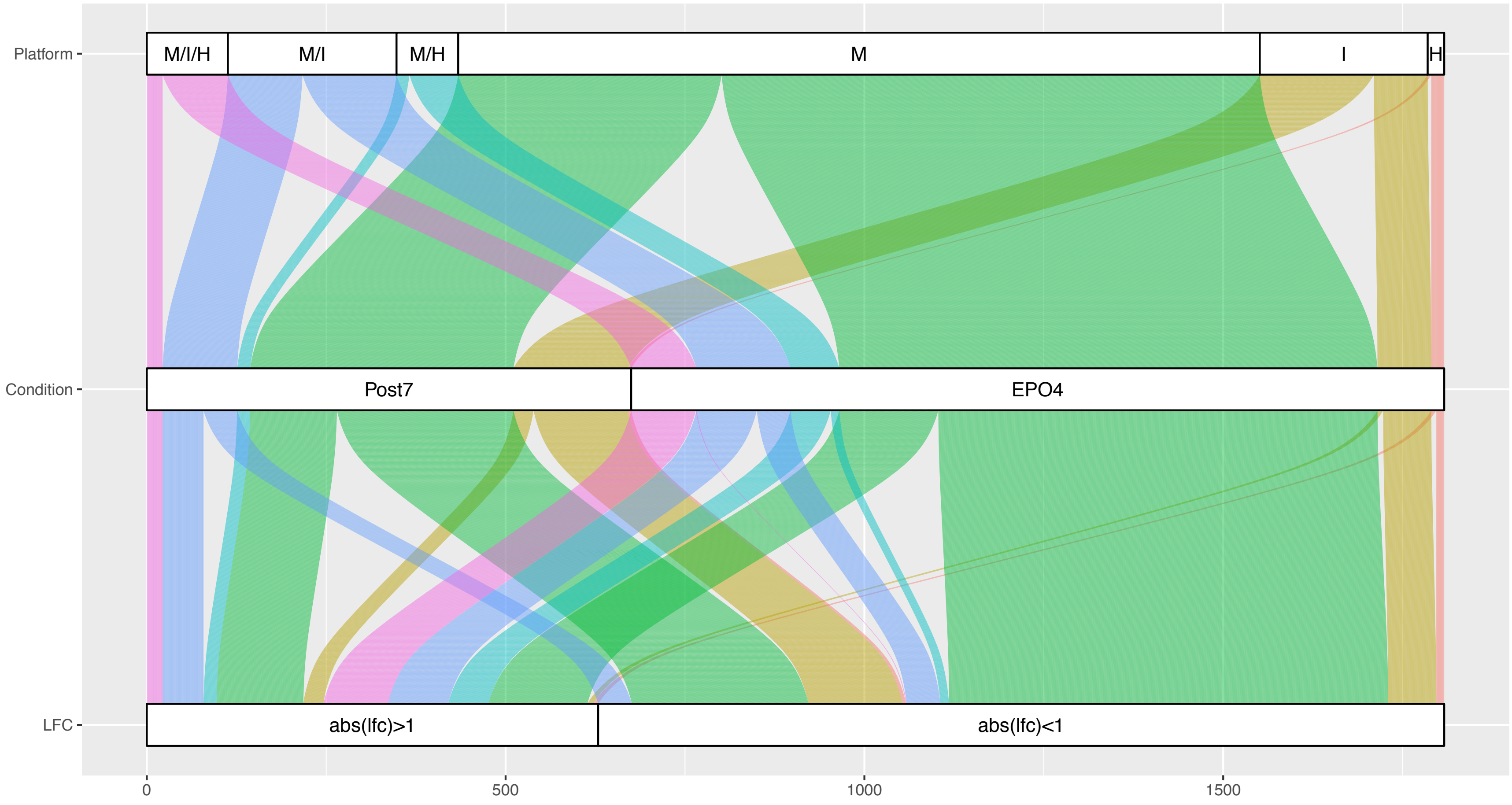

Supplementary Fig. S7. Sankey diagram showing the flow of the differentially expressed gene features stratified by platform, biological condition and absolute log_2-transformed fold changes. M/I/H: MGI RNA-seq/Illumina RNA-seq/HTA2.0; M/I: MGI RNA-seq/Illumina RNA-seq; M/H: MGI RNA-seq/HTA2.0; M: MGI RNA-seq; I: Illumina RNA-seq; and H: HTA2.0. abs(lfc): absolute log_2-transformed fold change. The colour coded band represents a detection platform or a combination of the detection platforms. The wider the band, the higher number of the identified features on a platform or across platforms. The x-axis represents the number of identified features captured on each platform. Note, for M/I/H, M/I, and M/H, that biological magnitude of the features

used for stratification is based on the MGI RNA-seq DGE results. Thirty-four identified non-protein coding transcript clusters on the GeneChip are removed for the purposes of cross-platform comparison

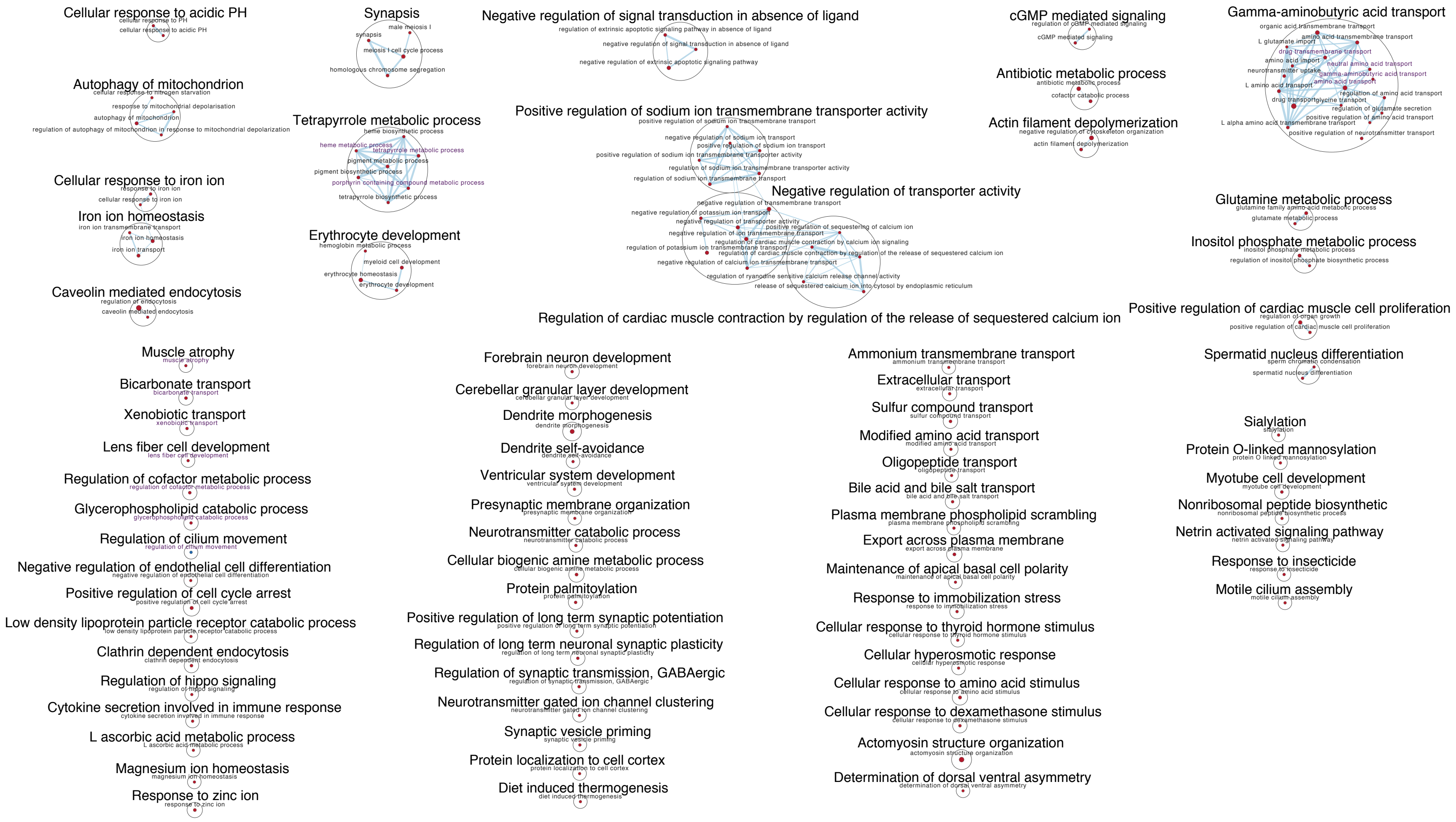

Supplementary Fig. S8. Biological network of the GeneChip dataset following Gene Ontology (biological process) gene set enrichment analysis in GSEA (v4.0.3) and visualisation in Cytoscape (3.8.0). Each circle (node) represents a gene set and two nodes are connected by lines (edges) indicating shared genes. The size of a node and width of an edge are proportional to the number of genes enriched in a gene set and the number of genes shared between gene sets, respectively. Gene sets that are similar were

annotated and clustered to form a biological theme using the AutoAnnotate App in Cytoscape. The most significantly enriched gene set is used to label a gene set cluster, defined by NES. Red node: gene set enriched in EPO4. Blue node: gene set negatively correlated with EPO4. Purple node label: top gene sets with NES >1.90. The enrichment map was created with pathway FDR<0.1, nominal P<0.05 and Jaccard Overlap coefficient >0.375 with combined constant k=0.5.

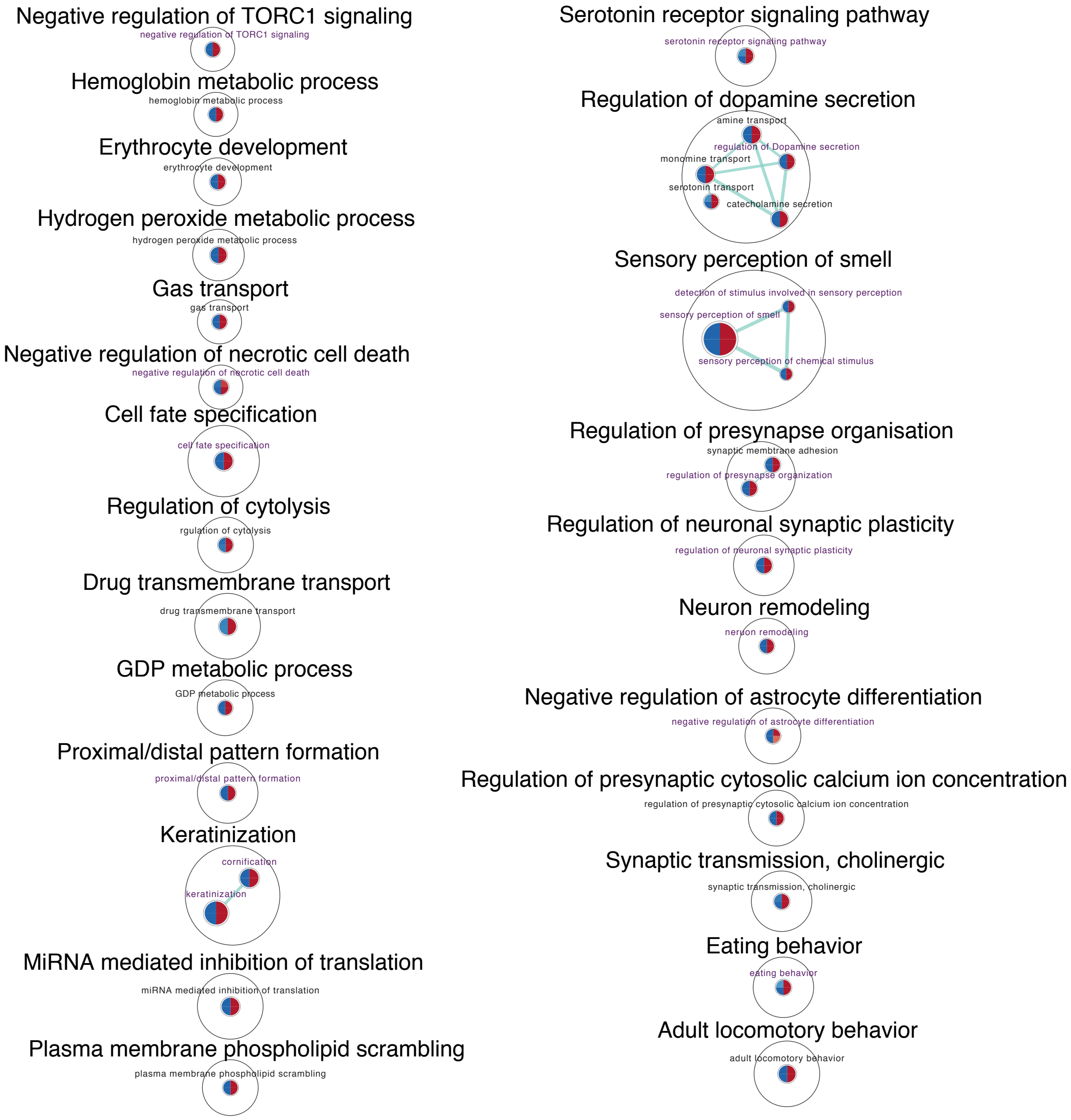

Supplementary Fig. S9. Biological network of the BeadChip datasets following Gene Ontology (biological process) gene set enrichment analysis in GSEA (v4.0.3) and visualisation in Cytoscape (3.8.0). Each circle (node) represents a gene set and two nodes are connected by lines (edges) indicating shared genes. The size of a node and width of an edge are proportional to the number of genes enriched in a gene set and the number of genes shared between gene sets, respectively. Gene sets that are similar were annotated and clustered to form a biological theme using the AutoAnnotate App in Cytoscape. The most significantly enriched gene set is used to label a gene set cluster, defined by NES. Red node: gene sets enriched in EPO4 and 5. Blue node: gene sets enriched in Post 7 and 8. Purple node label: top gene sets with NES >1.90. The enrichment map was created with pathway FDR<0.1, nominal P<0.05 and Jaccard Overlap coefficient >0.375 with combined constant k=0.5.

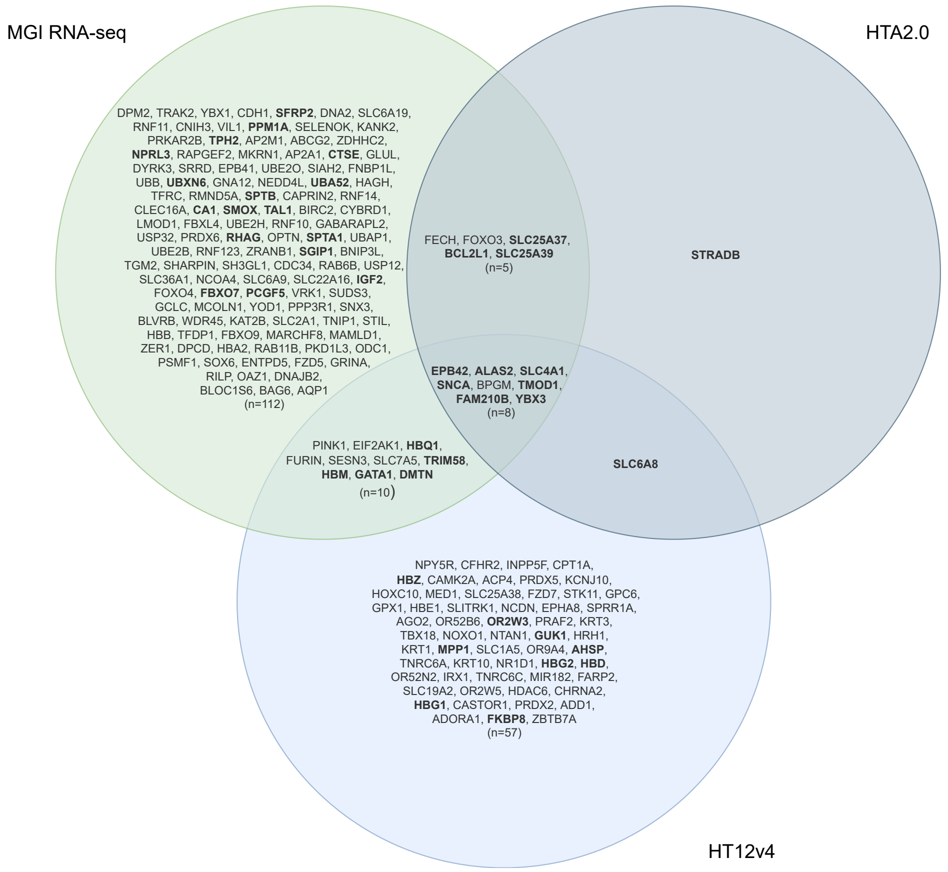

Supplementary Fig. S10. Genes differentially expressed and prioritised across the platforms and across the time points following the DGE and GSEA analyses. Genes highlighted in bold (n=43) were also significantly expressed following Illumina RNA-seq. The diagram was drawn using the online open source tool, draw.io (v15.0.4; https://github.com/jgraph/drawio).

Sample	% Aligned_Illumina	Reads in Million Aligned_Illumina	% Aligned_MGI	Reads in Million Aligned_MGI
**01**	94.1	109.9	96.0	157.9
**02**	94.8	130.7	95.8	210.5
**03**	94.8	116.2	95.1	154.9
**04**	94.1	135.6	95.5	148.7
**05**	94.3	113.8	95.6	151.5
**06**	94.7	114.8	95.7	168.1
**07**	95.0	117.7	95.9	152.9
**08**	94.9	101.8	96.2	179.9
**09**	94.8	116.1	96.0	160.8
**10**	95.4	112.0	91.5	162.2
**11**	95.7	102.9	91.7	161.3
**12**	95.6	96.4	91.5	166.3
**13**	95.3	92.7	94.8	184.0
**14**	95.6	81.2	92.6	170.1
**15**	95.3	82.9	92.7	173.7
**16**	95.0	115.6	91.5	167.0
**17**	94.7	129.0	92.7	166.5
**18**	94.8	123.8	93.2	189.3
**19**	95.2	129.9	91.9	179.7
**20**	94.2	97.3	92.4	209.8
**21**	95.2	105.4	89.4	183.0
**22**	94.6	109.5	92.2	216.4
**23**	95.3	105.8	97.3	247.0
**24**	95.4	103.1	95.9	196.5
**25**	95.2	99.1	93.0	182.6
**26**	93.7	117.3	92.0	175.1
**27**	94.4	117.1	91.7	165.7
**28**	95.0	112.7	92.4	164.3
**29**	94.7	157.0	90.9	178.5
**30**	94.2	102.9	90.1	209.8
**31**	94.8	122.0	91.1	237.6
**32**	95.2	115.5	91.2	311.3
**33**	-	-	89.6	212.3
**34**	95.2	111.2	92.8	230.4
**35**	95.0	102.5	96.1	215.2
**36**	95.2	107.5	96.5	281.5
**37**	95.7	98.6	91.6	225.0
**38**	95.5	102.1	97.5	191.6
**39**	95.7	118.1	96.9	311.7
**40**	-	-	97.3	195.6
**41**	94.5	97.6	97.1	303.1
**42**	95.3	103.8	97.7	216.2
**43**	95.3	96.1	97.2	190.5
**44**	95.2	102.9	97.3	212.0
**45**	95.5	100.7	97.8	225.4
**46**	95.0	115.9	95.6	215.7
**47**	95.1	116.6	96.1	202.4
**48**	95.8	123.2	96.3	207.0
**49**	94.8	113.5	95.4	194.9
**50**	94.3	100.1	95.8	183.6

**Average**	95.0	110.4	94.2	197.9

Supplementary Table S1. HISAT2 alignment against the reference genome assembly (GRCh38.p12) with Ensembl 94 annotation showing the overall alignment rates and the total reads in million on the Illumina (N=48) and MGI (N=50) sequencing platforms.

Sample	Total Reads in Million_Illumina	% CDS_ Exons_ Illumina	% Intron_ Illumina	Total Reads in Million_MGI	% CDS_ Exons_ MGI	% Intron_ MGI
**01**	103.5	72.2	10.3	151.6	49.3	40.1
**02**	127.6	71.1	10.1	201.7	52.1	37.4
**03**	123.9	74.1	8.4	174.3	53.5	36.6
**04**	110.2	75.7	10.7	188.5	60.2	29.9
**05**	107.3	75.1	6.6	206.9	58.7	30.2
**06**	96.6	72.8	9.9	206.2	59.1	30.7
**07**	108.7	76.0	8.2	194.5	59.6	30.1
**08**	111.8	75.1	8.1	199.3	63.8	26.5
**09**	110.0	73.0	11.1	185.9	59.1	31.3
**10**	106.8	73.0	8.2	175.9	63.0	26.1
**11**	98.4	82.4	5.2	147.4	51.4	38.6
**12**	92.1	80.8	6.0	142.0	48.6	40.8
**13**	88.3	83.4	4.8	144.8	48.5	40.7
**14**	77.7	84.3	5.3	160.8	51.5	38.5
**15**	79.0	73.0	9.0	146.6	52.1	37.1
**16**	109.8	71.5	8.0	173.1	53.7	35.7
**17**	122.2	71.1	10.4	154.4	59.3	30.9
**18**	117.4	70.8	9.9	148.3	56.1	33.9
**19**	123.6	72.5	11.0	147.9	63.3	28.0
**20**	91.7	70.7	10.2	152.1	61.1	29.1
**21**	100.4	76.4	8.0	157.6	46.4	43.5
**22**	100.8	79.3	6.8	160.9	51.2	38.6
**23**	103.6	76.2	6.9	152.9	48.1	41.9
**24**	98.4	80.6	6.9	154.3	57.0	32.8
**25**	94.4	72.8	8.9	176.5	50.1	39.5
**26**	148.7	71.3	11.0	165.2	51.9	37.8
**27**	110.0	69.6	11.6	193.9	49.3	40.4
**28**	110.5	74.0	8.4	163.5	51.0	40.8
**29**	107.1	75.8	8.8	199.4	46.8	44.4
**30**	96.9	71.2	9.6	240.3	49.1	38.7
**31**	105.9	71.1	11.4	169.7	48.4	41.7
**32**	115.6	73.7	8.3	161.1	53.9	35.4
**33**	-	-	-	151.9	46.2	41.9
**34**	100.9	80.1	7.4	151.8	57.4	33.4
**35**	97.4	73.8	7.6	162.3	59.3	30.6
**36**	97.5	75.4	9.1	188.9	54.8	35.9
**37**	102.3	73.9	10.0	216.4	51.0	39.1
**38**	94.4	77.1	8.4	284.0	44.7	45.9
**39**	113.0	79.8	7.9	190.3	47.9	43.0
**40**	-	-	-	213.8	51.8	37.9
**41**	91.6	71.2	10.9	271.5	42.3	46.4
**42**	92.3	71.2	9.9	206.1	42.8	48.5
**43**	98.9	71.0	10.1	186.9	43.5	46.3
**44**	98.0	78.7	7.6	301.9	47.7	41.9
**45**	96.2	71.7	9.4	190.4	48.9	40.3
**46**	110.0	71.8	9.1	294.4	54.7	35.2
**47**	107.6	73.5	8.5	211.2	53.9	35.8
**48**	110.9	72.8	9.0	185.2	48.7	40.2
**49**	118.0	83.5	5.8	206.3	54.6	34.6
**50**	94.4	72.0	8.6	220.5	45.1	44.3

**Average**	104.6	74.8	8.7	186.6	52.4	37.4

Supplementary Table S2. RSeQC read distribution including the total reads, % CDS_Exons tag counts and % intron tag counts aligned to the reference genome assembly (GRCh38.p12) with Ensembl 94 annotation on the Illumina (N=48) and MGI (N=50) sequencing platforms. CDS: coding sequences.

Sample	% Aligned_Illumina	Reads in Million Aligned_Illumina	% Aligned_MGI	Reads in Million Aligned_MGI
**01**	79.0	43.4	38.8	30.7
**02**	79.6	54	40.6	42.7
**03**	81.8	53.4	38.5	35.4
**04**	78.5	45.6	39.0	38.3
**05**	84.5	48.1	48.4	52.1
**06**	80.0	40.8	42.2	45.5
**07**	82.4	47.3	41.2	41.7
**08**	82.7	48.6	42.8	44.3
**09**	78.5	45.6	38.1	37.1
**10**	83.1	46.5	49.7	45.6
**11**	85.4	44	36.9	28.6
**12**	85.5	41.2	38.2	28.4
**13**	86.3	40	38.8	29.4
**14**	86.4	35.1	36.5	30.7
**15**	81.2	33.7	42.1	32.2
**16**	82.7	47.8	39.8	35.8
**17**	80.1	51.7	40.5	32.6
**18**	80.3	49.7	38.6	31.3
**19**	79.3	51.5	33.0	26.6
**20**	80.0	38.9	43.6	36.2
**21**	82.3	43.4	36.9	31.4
**22**	84.4	44.7	39.5	34.3
**23**	84.0	46	38.2	31.9
**24**	84.7	43.7	40.7	33.9
**25**	81.8	40.5	40.4	38.2
**26**	79.1	62.1	38.7	34.8
**27**	78.2	45.9	36.0	37.8
**28**	82.5	48.3	28.7	26.3
**29**	82.3	46.4	28.3	30.6
**30**	80.8	41.6	42.8	52.8
**31**	77.9	43.3	37.7	34.4
**32**	82.9	50.6	41.2	36.1
**33**	-	-	39.1	32.4
**34**	83.7	44.3	36.3	29.8
**35**	83.7	42.9	45.7	40.8
**36**	81.8	41.8	37.1	38.9
**37**	80.5	43.3	35.7	42.4
**38**	82.8	40.8	29.8	46.4
**39**	83.6	49.3	29.8	31.6
**40**	-	-	39.6	45.7
**41**	78.2	37.6	35.4	49.8
**42**	79.8	39	29.3	32.9
**43**	79.7	41.4	37.8	36.2
**44**	83.3	42.9	36.1	56.2
**45**	81.1	40.8	41.8	40.9
**46**	80.8	46.8	43.2	65.4
**47**	82.0	46.6	44.3	47.9
**48**	80.8	47.1	41.6	39.6
**49**	86.6	53.4	42.4	45
**50**	82.1	41.1	39.6	44.6
**Average**	81.9	45.1	38.8	38.3

Supplementary Table S3. Salmon alignment rates and reads in million following Illumina (N=48) and MGI (N=50) RNA-seq. Salmon indexing against Ensembl 94 annotated coding transcriptome.

		M/I/H	M/I	M/H	M	I	H
abs(lfc)>1	EPO4	86 (7.6%)	88 (7.8%)	54 (4.8%)	140 (12.4%)	8 (0.7%)	4 (0.4%)
	Post7	22 (3.3%)	57 (8.5%)	18 (2.7%)	121 (18.0%)	28 (4.2%)	0 (0.0%)
abs(lfc)<1	EPO4	1 (0.1%)	47 (4.2%)	11 (1.0%)	613 (54.4%)	67 (6.0%)	7 (0.6%)
	Post7	0 (0.0%)	47 (7.0%)	0 (0.0%)	246 (36.5%)	131 (19.4%)	4 (0.6%)

Supplementary Table S4. The number and percentages of identified features overlapped and unique across the biological conditions (EPO4 and Post7) and across the MGI RNA-seq (M), Illumina RNA-seq (I) and GeneChip (H) platforms. abs(lfc): absolute log_2-transformed fold change. For M/I/H, M/I and M/H, abs(lfc) grouping is based on the MGI RNA-seq DGE results of the gene features. 34 identified non-protein coding transcript clusters on the GeneChip are removed for the purposes of cross-platform comparison.

**Gene set**	**FDR**
**MGI RNA-seq (15,862 genes)**	
Enriched in Base2 (5)	
**E2F targets**	**0.067**
**G2M checkpoint**	**0.070**
**MTORC1 signaling**	**0.080**
**Glycolysis**	0.106
**Adipogenesis**	0.235
Enriched in EPO3 (2)	
**Heme metabolism**	0.137
**Pancreas beta cells**	0.238
Enriched in EPO4 (7)	
**G2M checkpoint**	**0.007**
**Heme metabolism**	**0.011**
**Reactive oxygen species pathway**	**0.012**
**Mitotic spindle**	**0.016**
**Protein secretion**	**0.038**
**Spermatogenesis**	**0.038**
**UV response up**	0.191
Enriched in Post7 (6)	
Fatty acid metabolism	0.197
MTORC1 signaling	0.206
MYC targets v2	0.211
Adipogenesis	0.234
**MYC targets v1**	0.242
Unfolded protein response	0.242
Enriched in Base1 (negatively correlated with Post7; 1)	
**Heme metabolism**	0.138
**Illumina RNA-seq (15,706 genes)**	
Enriched in Base2 (3)	
**G2M checkpoint**	
E2F targets	0.166
Unfolded protein response	0.179
	0.218
Enriched in EPO4 (1)	
**Heme metabolism**	
	**0.033**
Enriched in Base1(negatively correlated with EPO4; 1)	
**MYC targets v1**	
	**0.057**
Enriched in Post7 (1)	

Oxidative phosphorylation	0.212
**GeneChip HTA2.0 (21,137 genes)**	
Enriched in Base2 (22)	
**Glycolysis**	**0.003**
**E2F targets**	**0.010**
**Adipogenesis**	**0.013**
**G2M checkpoint**	**0.031**
**MTORC1 signaling**	**0.100**
**Notch signalling**	0.102
**Oxidative phosphorylation**	0.114
**Apoptosis**	0.115
**Mitotic spindle**	0.127
UV response up	0.142
Enriched in EPO3 (3)	
**Heme metabolism**	0.184
**Epithelial mesenchymal transition**	0.232
Estrogen response early	0.242
Enriched in EPO4 (6)	
**Heme metabolism**	**0.005**
**Hypoxia**	0.174
Xenobiotic metabolism	0.204
**Spermatogenesis**	0.218
**UV response up**	0.233
**Mitotic spindle**	0.239
Enriched in Base1 (negatively correlated with EPO4; 21)	
**P53 pathway**	**0.098**
**Allograft rejection**	0.119
**MYC target v2**	0.120
**Fatty acid metabolism**	0.120
**WNT beta catenin signaling**	0.141
Oxidative phosphorylation	0.141
Interferon gamma response	0.146
Unfolded protein response	0.147
Glycolysis	0.148
MYC targets v1	0.156
Enriched in Base1 (negatively correlated with Post7; 1)	
**Heme metabolism**	**0.017**

**Illumina Beadchip H12v4 (18,674 genes)**	
Enriched in EPO3 (7)	
**Heme metabolism**	**0.001**
**Angiogenesis**	**0.010**
**Epithelial mesenchymal transition**	**0.022**
**Myogenesis**	**0.031**
**Coagulation**	0.108
**KRAS signaling dn**	0.151
**Apical junction**	0.223
Enriched in EPO4 (1)	
**Heme metabolism**	**0.001**
Enriched in Base1 (negatively correlated with EPO4; 11)	
**E2F targets**	**0.033**
**MYC targets v2**	**0.043**
**MYC targets v1**	**0.086**
**Allograft rejection**	0.122
Unfolded protein response	0.156
**Fatty acid metabolism**	0.175
**G2M checkpoint**	0.189
**Peroxisome**	0.193
Oxidative phosphorylation	0.196
**MTORC1 signaling**	0.202
Enriched in EPO5 (1)	
**Heme metabolism**	**<0.001**
Enriched in Base1 (negatively correlated with EPO5, 3)	
**MYC targets v1**	0.197
**MYC targets v2**	0.229
**Allograft rejection**	0.222
Enriched in Post7 (1)	
**Peroxisome**	0.246
Enriched in Base1 (negatively correlated with Post7; 1)	
**Heme metabolism**	**0.003**
Enriched in Base1 (negatively correlated with Post8; 1)	
**Heme metabolism**	**0.004**

Supplementary Table S5. Summary of GSEA results exceeding FDR ≤ 0.25 in all Supplementary Data ets against the MSigDB hallmark gene sets. When the number of enriched gene sets (FDR≤0.25) exceeds 10, only the top 10 gene sets are included in the table. Enriched gene sets with FDR≤0.1 are highlighted in **bold** in the "FDR" column, while gene sets with nominal P value≤0.05 are highlighted in **bold** in the "Gene Set" column. Heme metabolism pathway results are marked in **red**.

Gene	baseMean	logFC	lfcSE	*s*-value	Condition	GSEA pathways (FDR < 0.1)	Reactome pathways (FDR < 0.05)
SLC25A37	212409.29	-1.76	0.16	0	Post7	transition metal ion homeostasis (EPO4); iron ion homeostasis (EPO4)	mitochondrial iron-sulfur cluster biogenesis (EPO4/Post7)
ALAS2	60117.99	-2.52	0.25	0	Post7	porphyrin-containing compound metabolic process (EPO4); transition metal ion homeostasis (EPO4); iron ion homeostasis (EPO4)	metabolism of porphyrins (EPO4/Post7)
CA1	7185.21	2.15	0.18	3.76E-25	EPO4	bicarbonate transport (EPO4)	erythrocytes take up oxygen and release carbon dioxide (EPO4/Post7); erythrocytes take up carbon dioxide and release oxygen (EPO4/Post7)
TFDP1	6221.33	1.80	0.15	1.06E-24	EPO4	N/A	transcriptional activity of SMAD2/SMAD3:SMAD4 heterotrimer (EPO4/Post7)
UBE2F	3258.00	1.12	0.09	4.82E-23	EPO4	N/A	neddylation (EPO4)
ALAS2	60117.99	2.49	0.25	5.08E-20	EPO4	porphyrin-containing compound metabolic process (EPO4); transition metal ion homeostasis (EPO4); iron ion homeostasis (EPO4)	metabolism of porphyrins (EPO4/Post7)
AP2A1	2565.40	1.28	0.12	1.19E-17	EPO4	N/A	MHC class II antigen presentation (EPO4); cargo recognition for clathrin-mediated endocytosis (EPO4/Post7)
RHAG	569.34	1.31	0.14	5.43E-16	EPO4	erythrocyte development (EPO4); erythrocyte	erythrocytes take up oxygen and release carbon dioxide

						homeostasis (EPO4); transition metal ion homeostasis (EPO4); iron ion homeostasis (EPO4); bicarbonate transport (EPO4)	(EPO4/Post7); erythrocytes take up carbon dioxide and release oxygen (EPO4/Post7); rhesus glycoproteins mediate ammonium transport (EPO4)
CUL4A	10140.15	1.00	0.10	1.07E-15	EPO4	N/A	neddylation (EPO4)
ANKRD9	1735.58	1.60	0.17	1.15E-15	EPO4	N/A	neddylation (EPO4)
ABCG2	909.90	1.61	0.18	1.40E-15	EPO4	N/A	metabolism of porphyrins (EPO4)

Supplementary Table S6. Top 10 genes resulted from the GSEA GO biological process, Reactome pathway and DESeq2 DGE analyses in the MGI RNA-seq datasets. LogFC: $\log_2$ fold change; lfcSE: standard error of $\log_2$ fold change; FDR: false discovery rate. N/A: not applicable. Genes are sorted by the *s*-value.

Gene	AveExpr	logFC	t	P.Value	Adj.P.Val	Condition	GSEA pathways (FDR < 0.1)	Reactome pathways (FDR < 0.05)
SLC6A8	6.49	2.49	13.95	1.35E-27	1.44E-23	EPO4	drug transmembrane transport (EPO5)	creatine metabolism (Post7/8)
TFDP1	8.24	1.57	12.54	3.36E-24	5.10E-21	EPO4	N/A	oxidative stress induced senescence (EPO4/5)
SIAH2	7.67	1.94	11.97	8.42E-23	5.96E-20	EPO4	N/A	amyloid fiber formation (EPO3/Post7)
SLC6A8	6.49	2.10	11.50	1.16E-21	1.15E-17	EPO5	drug transmembrane transport (EPO5)	creatine metabolism (Post7/8)
E2F2	8.99	1.91	10.26	1.28E-18	1.97E-16	EPO4	N/A	oxidative stress induced senescence (EPO4/5/Post7)
MAP2K3	10.04	1.54	9.50	9.02E-17	9.12E-15	EPO4	N/A	oxidative stress induced senescence (EPO4/5/Post7)
SNCA	9.98	2.29	9.14	6.96E-16	6.21E-14	EPO4	regulation of neuronal synaptic plasticity (Post7); regulation of dopamine secretion (Post8); regulation of presynapse organization (Post8); hydrogen peroxide metabolic process (Post7); adult locomotory behaviour (Post7)	amyloid fiber formation (Post7/8)
SNCA	11.32	-2.21	-9.37	1.86E-16	1.41E-13	Post7	regulation of neuronal synaptic plasticity (Post7); regulation of dopamine secretion Post8); regulation of	amyloid fiber formation (Post7/8)

							presynapse organization (Post8); hydrogen peroxide metabolic process (Post7); adult locomotory behaviour (Post7)	
TFDP1	8.18	1.14	9.07	1.01E-15	6.28E-13	EPO5	N/A	oxidative stress induced senescence (EPO4/5)
GUK1	11.61	-1.38	-8.74	6.38E-15	2.71E-12	Post7	GDP metabolic process (Post7)	interconversion of nucleotide di- and triphosphates (Post7/8)
AP2M1	8.48	1.03	8.33	5.77E-14	3.69E-12	EPO4	N/A	potential therapeutics for SARS (EPO4/5)
SIAH2	7.67	1.45	8.44	3.30E-14	1.13E-11	EPO5	N/A	amyloid fiber formation (EPO3/Post7)
FURIN	6.84	1.36	7.87	7.12E-13	3.74E-11	EPO4	N/A	potential therapeutics for SARS (EPO4/5)
TNRC6C	7.80	1.14	7.83	8.56E-13	4.38E-11	EPO4	N/A	oxidative stress induced senescence (EPO4/5/Post7/8)

Supplementary Table S7. Top 10 genes resulted from the GSEA GO biological process, Reactome pathway and limma DGE analyses in the BeadChip datasets. AveExpr: average expression values; LogFC: log_2 fold change; Adj.P.Val: BH adjusted *P*-value; FDR: false discovery rate. N/A: not applicable. Genes are sorted by the BH adjusted *P*-value.

Supplementary Data 1: Summary of the lfc values of the statistically significant common features present across MGI RNA-seq, Illumina RNA-seq and GeneChip HTA2.0 platforms at both EPO4 and Post7.

Supplementary Data 2: Lists of leading edge genes following the GSEA with the hallmark gene sets across all platforms and conditions.

Supplementary Data 3: Merged leading edge genes following the GSEA with the hallmark gene sets with the DGE genes identified across MGI RNA-seq, Illumina RNA-seq and GeneChip HTA2.0 platforms.

Supplementary Data 4: Merged leading edge genes following the GSEA with the hallmark gene sets with the DGE genes identified on the Illumina BeadChip platform.

Supplementary Data 5: Common gene features identified following MGI RNA-seq by merging GSEA leading edge genes and DGE genes across conditions.

Supplementary Data 6: Common gene features identified following GeneChip HTA2.0 analysis by merging GSEA leading edge genes and DGE genes across conditions.

Supplementary Data 7: Common gene features identified following Illumina BeadChip analysis by merging GSEA leading edge genes and DGE genes across conditions.

Supplementary Data 8: Common and platform-independent leading edge genes and pathways across MGI RNA-seq, GeneChip HTA2.0 and Illumina BeadChip platforms following GSEA and the DGE analyses.

Supplementary Data 9: Forty-three leading edge genes identified from one or more of the three platforms (MGI RNA-seq, GeneChip HTA2.0 and Illumina BeadChip platforms) overlapping with Illumina RNA-seq DGE genes across conditions.

Supplementary Data 10: Reactome pathways represented by the 43 GSEA leading edge genes along with the significantly altered Reactome entities and Reactome pathway FDR involved in these Reactome pathways.

Supplementary Data 11: Reactome pathway diagrams represented by the 43 GSEA leading edge genes.

Supplementary Data 12: Results of Reacome quantitative pathway analysis of the MGI RNA-seq data.

Supplementary Data 13: Results of Reacome quantitative pathway analysis of the Illumina RNA-seq data.

Supplementary Data 14: Results of Reacome quantitative pathway analysis of the GeneChip data.

Supplementary Data 15: Results of Reacome quantitative pathway analysis of the BeadChip data.

Supplementary Data 16: Candidate gene lists and associated pathways in response to EPO following GSEA, Reactome and DGE analyses in the MGI RNA-seq and Illumina BeadChip datasets.
